# Synthesis and Evaluations of Novel Apocynin Derivatives as Anti-Glioma Agents

**DOI:** 10.3389/fphar.2019.00951

**Published:** 2019-09-03

**Authors:** Tao Yang, Da-Wei Zang, Wei Shan, An-Chen Guo, Jian-Ping Wu, Yong-Jun Wang, Qun Wang

**Affiliations:** ^1^Department of Neurology, Beijing Tiantan Hospital, Capital Medical University, Beijing, China; ^2^China National Clinical Research Centre for Neurological Diseases, Beijing Tiantan Hospital, Capital Medical University, Beijing, China; ^3^Beijing Institute for Brain Disorders, Capital Medical University, Beijing, China; ^4^Advanced Innovation Center for Human Brain Protection, Capital Medical University, Beijing, China

**Keywords:** apocynin derivatives, blood-brain barrier, anti-glioma, apoptosis, NF-κB activation

## Abstract

Apocynin (4-hydroxy-3-methoxyacetophenone) is a natural polyphenolic compound with multiple biological activities. In the present study, a series of apocynin derivatives were designed and synthesized. The *in silico* ADMET prediction, blood–brain barrier (BBB) penetration assay, anti-NADPH oxidase activity, reactive oxygen species (ROS) levels, and anti-glioma effects of these apocynin derivatives were evaluated. The anti-glioma mechanisms of candidate compounds were studied by ﬂow cytometer and Western blot. The results showed that D31 exhibited higher BBB penetration, increased ROS generations and significant anti-glioma effects both *in vitro* and *in vivo*. Further studies showed that D31 inhibited the activations of NF-κB pathway. Overall, our data demonstrated that D31 inhibited growth and induced apoptosis of glioma, which might be caused by ROS-related NF-κB activation. The current study suggested that D31 could be further explored for its potential use in anti-glioma therapy.

## Introduction

Gliomas are the most common malignant brain tumors and remain one of the most challenging types of cancer to treat. The prognosis of patients with malignant glioma is extremely poor despite advances in malignant glioma treatment in recent years (Bush et al., 2017). Temozolomide (TMZ) is the major chemotherapeutic drug for malignant glioma treatment. However, glioma cells quickly develop TMZ resistance, and the long-term clinical benefits of TMZ are poor (He et al., 2019). Thus, there is an urgent need to search novel anti-glioma agents that can improve therapeutic benefit and prolong survival of malignant glioma patients.

Natural products are significant sources of novel drug discovery, especially the anticancer drugs. Apocynin (4-hydroxy-3-methoxy-acetophenone) is a natural polyphenolic compound isolated from a variety of plant sources, including *Apocynum cannabinum, Picrorhiza kurroa*, and so on (‘t Hart et al., 2014). The pharmacological activities and action mechanisms of apocynin are extensively studied (Hou et al., 2019). Similar to other polyphenolic compounds, apocynin has been shown to have multiple pharmacological effects, such as anti-oxidant, anti-inflammation, and anticancer effects (Ma et al., 2018). Apocynin has been proven to be an efficient nicotinamide adenine dinucleotide phosphate (NADPH) oxidase (NOX) inhibitor in many cell and animal models (Qin et al., 2017; Min et al., 2018; Du et al., 2019) and has been widely used as a standard NOX inhibitor for research purposes. Recently, NOX family members are found to play critical roles in human cancers (Lambeth, 2004; Burtenshaw et al., 2017). It was shown that apocynin inhibited cancer cell proliferations *via* down-regulating cyclin D1 and inhibiting Rac1 phosphorylation, one component of the NOX complex (Suzuki et al., 2013).

Our previous study showed that apocynin attenuated cerebral ischemia-induced oxidative damage in gerbils and exhibited good neuroprotective effects (Wang et al., 2006). Our result also showed that apocynin was able to cross the blood–brain barrier (BBB) and distributed in the brain tissues (Wang et al., 2008). However, the BBB permeation rate of apocynin is low, and the amount of apocynin in the brain tissue is <8% of that present in the blood. Also, the stability of apocynin is poor due to the phenolic hydroxyl group in the structure. Recently, the anti-inflammatory activities of apocynin attract wide attention, and many apocynin derivatives have been designed and synthesized to improve the anti-inflammatory activity of apocynin (Paracatu et al., 2016; Zhang et al., 2017a). However, structure modifications of apocynin were seldomly carried out to explore more promising candidate for the treatment of malignant gliomas. In the present study, several novel apocynin derivatives are synthesized to enhance the BBB penetration ability and improve the stability of apocynin. Also, the anti-glioma effects of the candidate apocynin derivatives are evaluated.

## Materials and Methods

### General

All chemicals used were commercially available. Apocynin (D1, 98% purity) was purchased from Sigma Chemicals Co. (St. Louis, MO, USA). K_2_CO_3_,CHCl_3_, benzyl bromide, dimethylformamide (DMF), CH_3_CN, NaHCO_3_, Br_2_, aniline para-fluoroaniline, 2,4-dimethoxyaniline, 2,4-difluoroaniline, and 4-chloroaniline were purchased from Sinopharm Chemical Reagent Co., Ltd (Beijing, China). Proton nuclear magnetic resonance (^1^H NMR) and carbon nuclear magnetic resonance (^13^C NMR) spectra were recorded at on a Bruker Avance III 500MHz NMR Spectrometer (Bruker, Switzerland). Chemical shifts (δ) and coupling constants (J) were expressed in ppm and Hz, respectively.

Human glioma U87 cells, U251 cells, and rat C6 glioma cells were purchased from Chinese Academy of Medical Sciences (Beijing, China). Dulbecco’s Modified Eagle Medium (DMEM), fetal bovine serum (FBS), and trypsin were purchased from invitrogen (Carlsbad, CA, USA). Methylthiazolyldiphenyl-tetrazolium bromide (MTT) and phosphate-buffered saline (PBS) were purchased from Gibco BRL Co., Ltd. (Grand Island, New York, USA). Cells were cultured in DMEM supplemented with 10% FBS in a 37°C incubator. Cells were washed with PBS and harvested by trypsinization.

### Chemistry

**Scheme 1:**
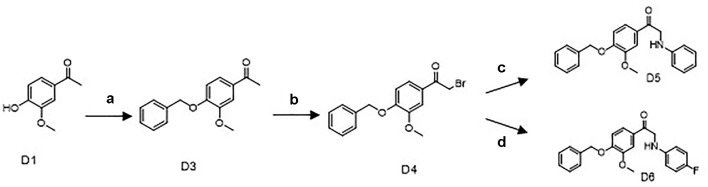
Synthetic routes of the apocynin derivatives D5 and D6. a) benzyl bromide, K_2_CO_3_, Dimethylformamide, 40°C, 4 h; b) Br_2_, CHCl_3_, r.t., 2 h; c) aniline, C H_3_CN, NaHCO_3_, r.t., 12 h; d) para-fluoroaniline, CH_3_CN, NaHCO_3_, r.t., 12 h.

**Scheme 2:**
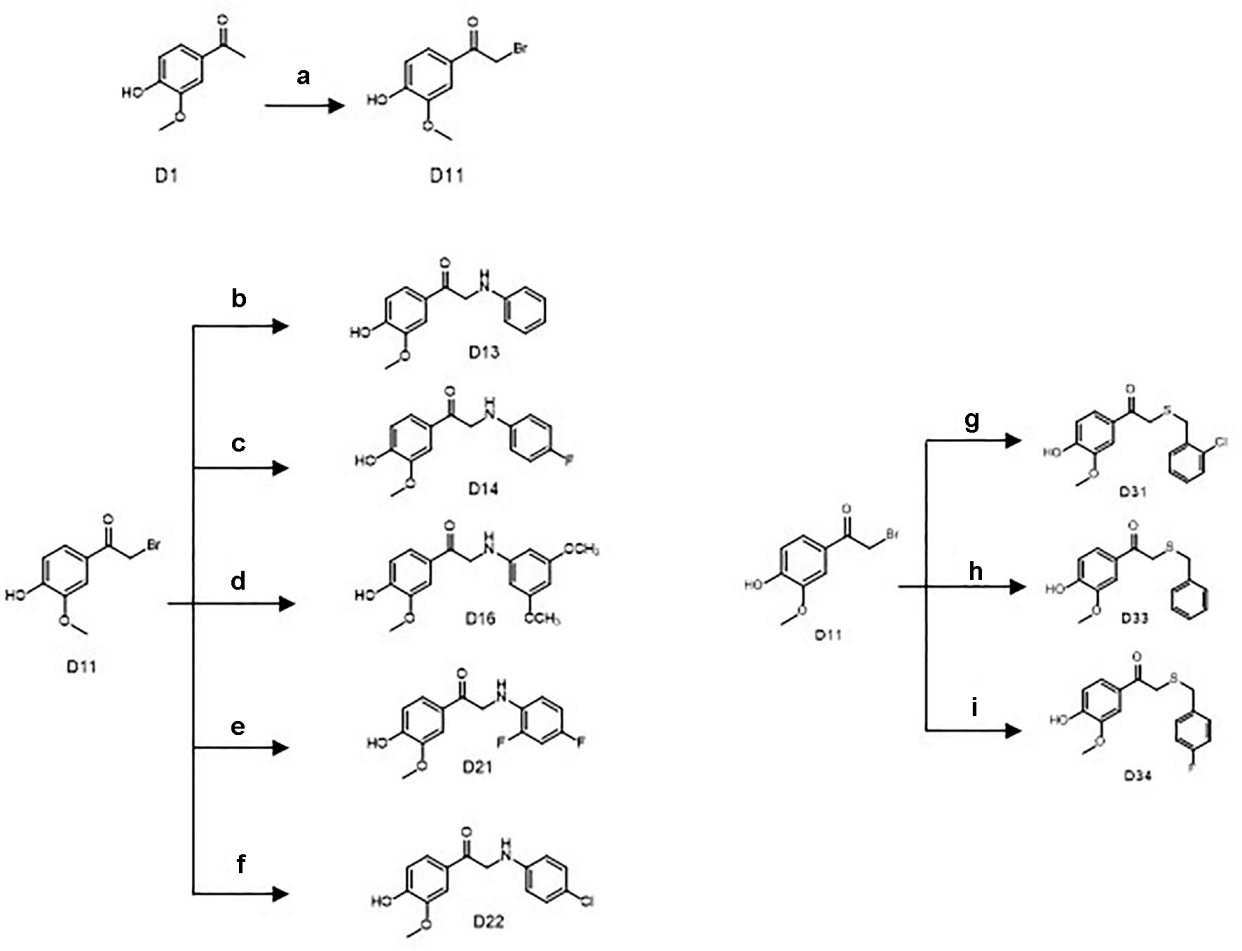
Synthetic routes of the apocynin derivatives (D13, D14, D16, D21, D22, D31, D33, and D34). a) CuBr_2_, ethyl acetate, r.t., 5 h; b) aniline, dimethylformamide (DMF), NaHCO_3_, r.t., 3 h; c) para-fluoroaniline, DMF, NaHCO_3_, r.t., 3 h; d) 2,4-dimethoxyaniline, DMF, NaHCO_3_, r.t., 3 h; e) 2,4-difluoroaniline, DMF, NaHCO_3_, r.t., 3 h; f) 4-chloroaniline, DMF, NaHCO_3_, r.t., 3 h; g) 2-chlorobenzyl mercaptan, dichloromethane (DCM), triethylamine (TEA), r.t., 12 h; h) benzyl mercaptan, DCM, TEA, r.t., 12 h; i) 4-fluorobenzyl mercaptan, DCM, TEA, r.t., 12 h.

**Figure 1:**
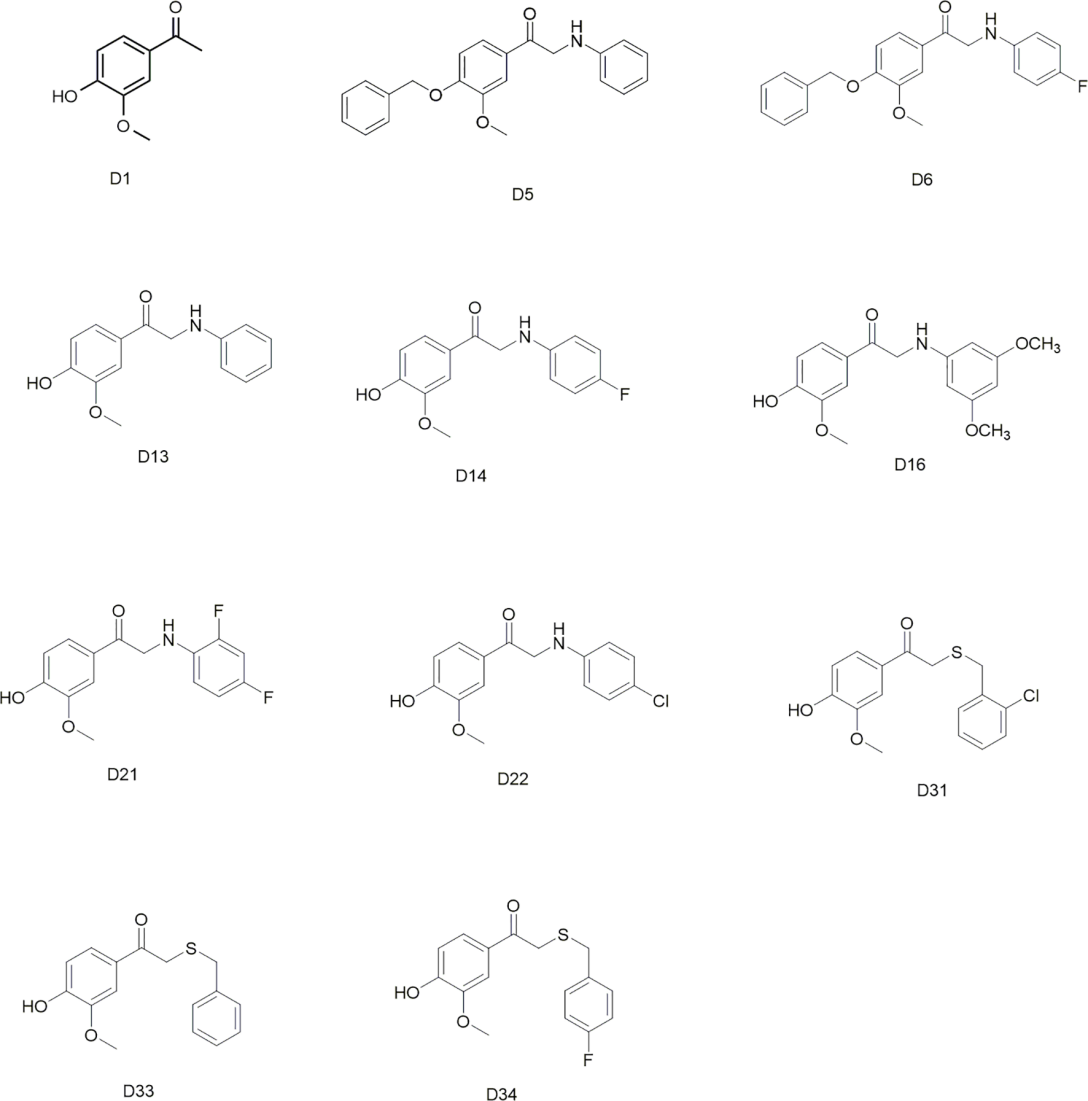
Structures of the synthesized apocynin derivatives.

Commercially available D1 was selected as the starting material. The synthetic protocols for the newly designed derivatives were outlined in **Schemes 1** and **2**. All the novel apocynin derivatives were fully characterized by ^1^H NMR, ^13^C NMR, and high-resolution mass spectrometry (HRMS) measurements, and the analytical and spectroscopic data for these derivatives were reported in the **Supporting Information**. The structures of the newly synthesized apocynin derivatives are shown in **Figure 1**.

#### Synthesis of the Key Intermediate D4

D1 (6.7 g, 40 mmol), K_2_CO_3_ (16.6 g, 120 mmol), and 30-ml DMF were mixed and stirred at 40°C for 10 min, then 5.2ml benzyl bromide (D2) was added to react for 4 h at 40°C. After cooling to room temperature, the reaction mixture was filtered and washed with ice-cold water to afford 12 g D3 as a white powder (97.7% yield, 98% purity) (**Scheme 1**).

The key intermediate D4 was synthesized from D3 by bromo substitution reactions. Compound D3 (2.46 g, 8 mmol) was first dissolved in 50ml CHCl_3_. A solution of Br_2_ (0.5 ml, 8 mmol) in 50 ml CHCl_3_ was added dropwise, and the reaction mixture was stirred at room temperature for 2 h. After being washed with saturated sodium thiosulfate and saturated sodium chloride, the product was purified by silica gel chromatography to afford 1.4 g of D4 as a white powder (52.4% yield, 98% purity).

#### Synthesis of 1-(4-(Benzyloxy)-3-Methoxyphenyl)-2-(Phenylamino)Ethan-1-One (D5)

D4 (334 mg, 1 mmol) and 0.1 ml aniline (1.1 mmol) in the presence of 5ml CH_3_CN and 168-mg NaHCO_3_ were reacted at room temperature for 12 h. After quenching reaction with water, the reaction mixture was extracted with ethyl acetate, washed with saturated sodium chloride, and purified by silica gel chromatography, to afford 170 mg of D5 as a deep yellow powder. The purity of D5 was 98% with yield up to 49%. ^1^H NMR (500 MHz, CDCl_3_) δ3.97 (s,3H), 4.56 (s,2H), 5.25 (s,2H), 6.71 (d,J = 8.0Hz,1H), 6.75 (m,1H), 6.95 (d,J = 8.0Hz,1H), 7.22 (m,2H), 7.33 (m,1H), 7.39 (m,1H), 7.44 (m,2H), 7.58 (m,2H); ^13^C NMR (500 MHz, CDCl_3_) δ50.0, 56.4, 71.0, 110.6, 112.5, 113.2, 117.9, 122.2, 127.4, 128.4, 128.5, 128.9, 129.6, 136.3, 147.4, 150.1, 153.2, 193.8.

#### Synthesis of 1-(4-(Benzyloxy)-3-Methoxyphenyl)-2-((4-Fluorophenyl)Amino) Ethan-1-One (D6)

The same treatment of D4 (334 mg, 1 mmol) with 0.1 ml para-fluoroaniline (1.1 mmol) in the presence of NaHCO_3_ and CH3CN afforded 180 mg of D6 as a light yellow powder. The purity of final product was 98% with yield up to 51.9% (**Scheme 1**). ^1^H NMR (500 MHz, CDCl_3_) δ3.95 (s,3H), 4.53 (s,2H), 5.26 (s,2H), 6.66 (m,2H), 6.93 (m,3H), 7.33 (m,1H), 7.39 (m,3H), 7.44 (m,3H), 7.56 (m,2H); ^13^C NMR (500 MHz, DMSO-d_6_) δ50.59, 56.33, 70.55, 111.14, 113.08, 113.91, 115.69, 115.87, 123.00, 128.54, 128.70, 128.84, 129.16, 137.16, 145.55, 149.57, 152.96, 154.23, 156.06, 195.09.

#### Synthesis of 1-(4-Hydroxy -3-Methoxyphenyl)-2-(Phenylamino)Ethan-1-One (D13)

Another key intermediate D11 was yielded by bromo substitution reactions of D1. D1 (2.7 g, 16 mmol), and CuBr_2_ (7.0 g, 28 mmol) in 50 ml ethyl acetate were mixed and refluxed for 5 h. The reaction mixture was then cooled, filtered, and washed with saturated sodium thiosulfate, saturated NaHCO_3_, and saturated sodium chloride (**Scheme 2**). After decompression drying and silica gel chromatography purification, 2.2 g of D11 as a white powder was afforded (56% yield, 98% purity).

D11 (370 mg, 1.5 mmol) and 0.2-ml aniline (2.0 mmol) in the presence of 5-ml *N*,*N*-Dimethylformamide and 252 mg NaHCO_3_ were reacted at room temperature for 3 h. After quenching the reaction with water, the reaction mixture was extracted with ethyl acetate, washed with saturated sodium chloride, and purified by silica gel chromatography to afford 190 mg of D13 as a yellow powder. The purity of D13 was 98% with yield up to 49.3%. ^1^H NMR (500 MHz, DMSO-d_6_) δ3.84 (s,3H), 4.56 (d,J = 5.0 Hz, 2H), 5.73 (s,1H), 6.54 (t,1H), 6.66 (d,J = 8.0 Hz, 2H), 6.88 (d,J = 8.0 Hz, 1H), 7.07 (t,2H), 7.53 (br.s.,1H), 7.64 (br.d., J = 8.0 Hz, 1H), 10.01 (s,1H); ^13^C NMR (500 MHz, DMSO-d_6_) δ49.92, 56.33, 111.72, 113.14, 115.69, 116.69, 123.42, 127.53, 129.45, 148.23, 148.83, 152.67, 195.35.

#### Synthesis of 2-((4-Fluorophenyl)amino)-1-(4-Hydroxy-3-Methoxyphenyl)Ethan-1-One (D14), 2-((3,5-Dimethoxyphenyl)Amino)-1-(4-Hydroxy-3-Methoxyphenyl)Ethan-1-One (D16), 2-((2,4-Difluorophenyl)Amino)-1-(4-Hydroxy-3-Methoxyphenyl)Ethan-1-One (D21), and 2-((4-Chlorophenyl)Amino)-1-(4-Hydroxy-3-Methoxyphenyl)Ethan-1-One (D22)

The same treatment of D11 (370 mg, 1.5 mmol) with 0.2ml para-fluoroaniline, 0.2ml 2,4-dimethoxyaniline, 0.2ml 2,4-difluoroaniline, or 0.2 ml 4-chloroaniline in the presence of NaHCO_3_ and CH_3_CN afforded 200 mg D14 (48.5% yield, yellow powder), 210mg D16 (44.2% yield, gray powder), 240mg D21 (54.6% yield, yellow powder), and 160-mg D22 (55.0% yield, light yellow powder)(**Scheme 2**). The purities of D14, D16, D21, and D22 were all >98%. D14: ^1^H NMR (500 MHz, DMSO-d_6_) δ3.86 (s,3H), 4.56 (s,2H), 5.73 (s,1H), 6.68 (m,2H), 6.92 (m,3H), 7.54 (d,J = 2.0 Hz, 1H), 7.65 (dd,J = 2.0, 8.0 Hz,1H), 10.03 (s,1H); ^13^C NMR (500 MHz, DMSO-d_6_) δ50.4356.37, 111.80, 113.89, 115.71, 115.86, 123.42, 127.55, 145.60, 148.25, 152.70, 154.14, 155.97, 195.28; D16: ^1^H NMR (500 MHz, DMSO-d_6_) δ3.64 (s,6H), 3.84 (s,3H), 4.53 (s,2H), 5.75 (s,1H), 5.89 (s,2H), 6.88 (d,J = 8.0 Hz, 1H), 7.52 (br.s.,1H), 7.64 (br.d.,J = 8.0 Hz,1H), 10.03 (s,1H); ^13^C NMR (500 MHz, DMSO-d_6_) δ50.00, 55.41, 56.33, 89.41, 92.07, 111.72, 115.67, 123.51, 127.49, 148.24, 150.61, 152.69, 161.77, 195.22; D21: ^1^H NMR (500 MHz, DMSO-d_6_) δ3.84 (s,3H), 4.60 (d,J = 5.5 Hz, 2H), 5.34 (s,1H), 6.70 (m,1H), 6.81 (m,1H), 6.87 (d,J = 8.0 Hz, 1H), 7.08 (m,1H), 7.50 (br.s.,1H), 7.60 (dd,J = 2.0, 8.0 Hz, 1H), 10.03 (s,1H); ^13^C NMR (500 MHz, DMSO-d_6_) δ49.70, 56.17, 103.77, 111.15, 113.08, 115.54, 123.29, 127.12, 133.69, 148.08, 150.50, 152.64, 153.47, 194.71; D22: ^1^H NMR (500 MHz, DMSO-d_6_) δ3.84 (s,3H), 4.57 (s,2H), 6.68 (d,J = 8.0 Hz, 1H), 6.89 (d,J = 8.0 Hz,1H), 7.08 (d,J = 8.0 Hz, 1H), 7.52 (br.s.,1H), 7.62 (br.d.,1H), 10.03 (s,1H); ^13^C NMR (500 MHz, DMSO-d_6_) δ49.57, 55.99, 111.43, 114.15, 115.34, 119.53, 123.06, 127.10, 128.71, 147.49, 147.86, 152.35, 194.61.

#### Synthesis of 2-((2-Chlorobenzyl)thio)-1-(4-Hydroxy-3-Methoxyphenyl)Ethan-1-One (D31)

D11 (370 mg, 1.5 mmol) and 0.3-ml 2-chlorobenzyl mercaptan (2.0 mmol) in the presence of 5-ml dichloromethane and 0.3 ml triethylamine were reacted at room temperature for 12 h. After quenching the reaction with water, the reaction mixture was extracted with ethyl acetate, washed with saturated sodium chloride, and purified by silica gel chromatography to afford 200 mg D31 as a white powder. The purity of D31 was 98% with the yield up to 41.4%. ^1^H NMR (500 MHz, DMSO-d_6_) δ3.83 (s,3H), 3.85 (s,2H), 3.90 (s,2H), 7.31 (m,2H), 7.46 (m,3H), 7.52 (dd, J = 2.0, 8.0 Hz, 1H), 10.06 (s,1H); ^13^C NMR (500 MHz, DMSO-d_6_) δ33.41, 36.58, 55.94, 111.98, 115.23, 124.05, 127.27, 127.44, 129.28, 129.91, 131.54, 133.41, 135.74, 147.85, 152.31, 193.42.

#### Synthesis of 2-(Benzylthio)-1-(4-Hydroxy-3-Methoxyphenyl)Ethan-1-One (D33), 2-((4-Fluorobenzyl)Thio)-1-(4-Hydroxy-3-Methoxyphenyl)Ethan-1-One (D34)

The same treatment of D11 (370 mg, 1.5 mmol) with 0.3 ml benzyl mercaptan or 0.3 ml 4-fluorobenzyl mercaptan in the presence of 5 ml dichloromethane and 0.3 ml triethylamine afforded 220 mg D33 (50.1% yield, white powder) and 160 mg D34 (52.3% yield, white powder). The purity of D33 and D34 were > 98%. D33: ^1^H NMR (500 MHz, DMSO-d_6_) δ3.78 (s,2H), 3.84 (s,2H), 3.85 (s,3H), 6.88 (d,J = 8.0 Hz, 1H), 7.28 (m,1H), 7.35 (m,4H), 7.49 (br.s.,1H), 7.53 (br.d., J = 8.0 Hz,1H), 10.03 (s,1H); ^13^C NMR (500 MHz, DMSO-d_6_) δ35.99, 36.55, 56.30, 112.33, 115.57, 124.42, 127.61, 127.68, 129.05, 129.67, 138.58, 148.22, 152.63, 193.83; D34: ^1^H NMR (500 MHz, DMSO-d_6_) δ3.77 (s,2H), 3.84 (s,2H), 3.85 (s,3H), 6.88 (d,J = 8.0 Hz,1H), 7.17 (t,2H), 7.39 (m,2H), 7.48 (br.s.,1H) 7.53 (br.d.J = 8.0Hz,1H), 10.03 (s,1H); ^13^C NMR (500 MHz, DMSO-d_6_) δ35.11, 36.48, 56.29, 112.32, 115.57, 115.71, 115.88, 124.43, 127.64, 131.56, 134.87, 148.22, 152.65, 160.87, 162.81, 193.82.

### *In Silico* ADMET Prediction

Descriptors related to absorption, distribution, metabolism, excretion, and toxicity (ADMET) properties of the compounds were predicted using BIOVIA Discovery Studio 2016 (Accelrys Software Inc.). A series of parameters including absorption level, lipophilicity descriptor (AlogP98), BBB level, cytochrome P450 2D6 (CYP2D6) prediction, hepatotoxic prediction, plasmatic protein binding (PPB) prediction, polar surface area (PSA-2D), and solubility level were calculated. AlogP98 is for predicting atom-based molecular hydrophobicity or lipophilicity. PSA-2D is for predicting drug transport properties. Absorption level is for predicting intestinal absorption after being orally administrated in human (0, good; 1, moderate; 2, low; 3, very low). The BBB model is for predicting penetration of drugs across the BBB after being orally administrated (0, very high penetrant; 1, high; 2, medium; 3, low; 4, undefined). CYP2D6 prediction is for predicting the inhibitory effects of drugs on CYP2D6 using the cutoff Bayesian score of 0.161. Hepatotoxicity is for predicting the potential human hepatotoxicity. The PPB model is for predicting the plasma proteins bound ability of drugs (highly bound, ≥90% bound, using the cutoff Bayesian score of −2.209). Solubility level is for predicting drug solubility of in water at 25°C (0, extremely low; 1, very low; 2, low; 3, good; 4, optimal; 5, too soluble).

### BBB Penetration Assay *In Vitro*

#### Culture of Primary Rat Brain Microvascular Endothelial Cells and Astrocytes

**Figure 2:**
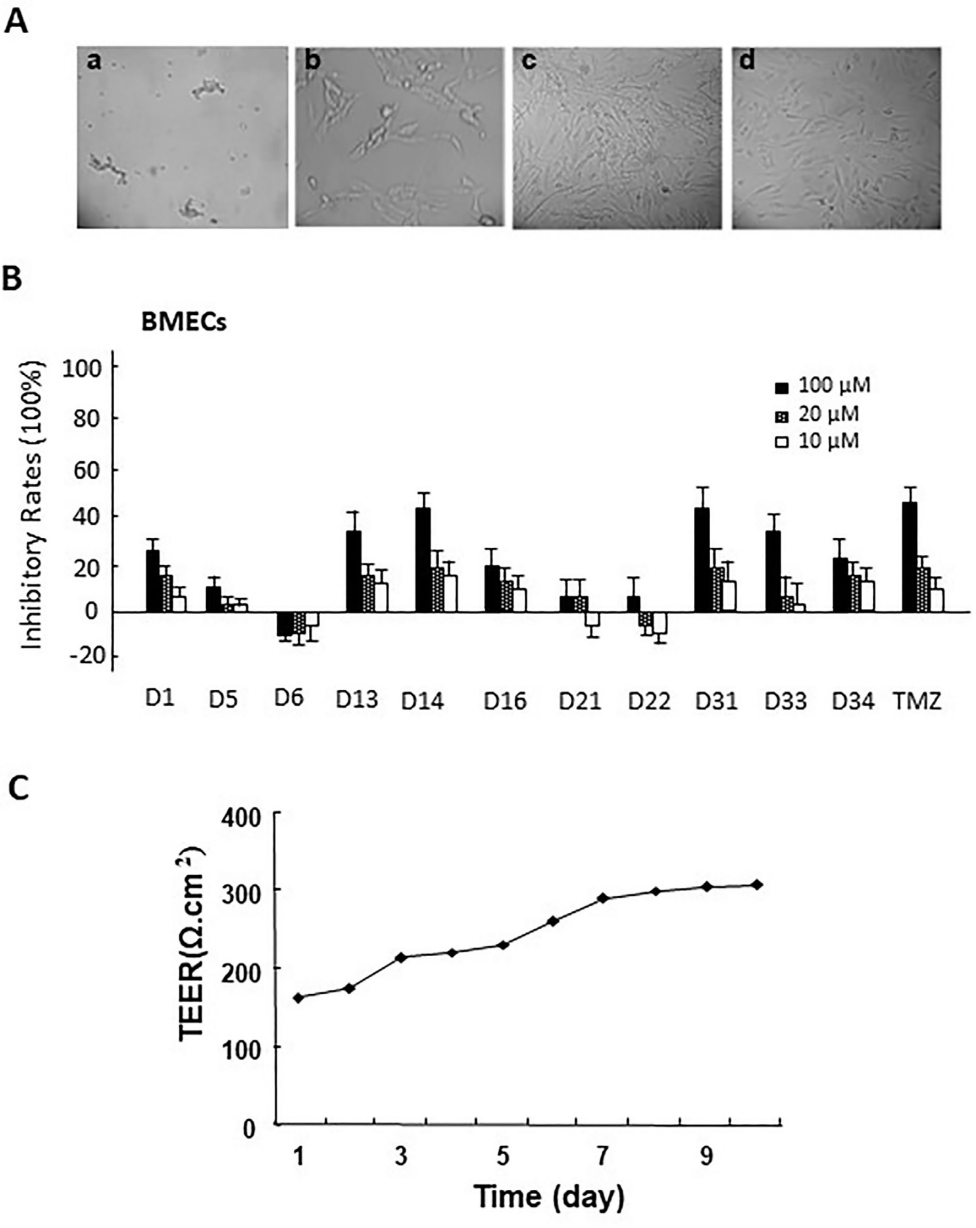
**(A)** The morphology of primary rat brain microvascular endothelial cells (BMECs) and astrocytes. a. microvascular fragment (×10); b. brain microvascular endothelial cells (×40); c. the morphology of primary astrocytes (×10); d. the morphology of passage 2 astrocytes (×10). **(B)** The cytotoxicity of apocynin derivatives on rat BMECs. **(C)** The TEER of BBB model co-cultured *via* rat BMECs and astrocytes at different time points.

Rat brain microvascular endothelial cells (BMECs) were separated from the cerebral cortex of 18-day-old male Sprague-Dawley rats as described previously (Biegel et al., 1995). Briefly, the cerebral cortical tissues were dissected from meninges after the rats were sacrificed, then the tissues were homogenated in physiological buffer solution (pH 7.4). After centrifugation, the pellets were collected, and the microvascular segments were then digested with 0.1% collagenase II at 37°C for 1 h. The suspension was filtered and then incubated in ECM supplemented with 5% FBS, 1% of penicillin and streptomycin, and 1% endothelial cell growth supplement (ECGS) in a humidified atmosphere with 5% CO_2_ at 37°C until the BMECs exhibited a typical “spread of pebble” growth pattern (**Figure 2A**). The second passage was used for co-culture of BBB cellular models.

Astrocytes were separated from the cerebral cortex of newborn Sprague-Dawley rats (1–2 days) as described previously (Jeliazkova-Mecheva and Bobilya, 2003). The brain tissues (removal of the meninges and blood vessel) were minced and mechanical homogenized and then digested with 0.25% trypsin-EDTA at 37°C for 10 min. After being filtered through a 75μm nylon mesh, the suspension was cultured in a culture flask containing DMEM supplemented with 10% FBS and 1% penicillin and streptomycin in a humidified atmosphere of 5% CO_2_ at 37°C until the astrocytes exhibited polygonal growth pattern (**Figure 2B**).

#### Cytotoxicity of Apocynin Derivatives on Rat BMECs

Rat BMECs were collected and seeded into 96-well culture plates at a density of 7.5 × 10^3^ cells/well overnight. The cells were treated with different concentrations of the compounds (10, 20, and 100 μM) for 24 h, respectively. Then, 20-μl MTT (5 mg/ml) in fresh medium was added to each well and incubated for another 4 h. Finally, 150μl dimethyl sulfoxide (DMSO) was added to dissolve formazan crystals, and the absorbance was detected at 490 nm in a multiwall-plate reader (BioTek ELx800).

#### Co-Culture of Rat BMECs and Astrocytes as BBB Cell Model

The *in vitro* BBB cell model was constructed in the Transwell 24well plate (0.4 mm pore size, Millipore, USA). Before the experiment, the transwell filters were coated with matrigel matrix. The isolated rat astroglias were seeded onto one side of polycarbonate inserts (5 × 10^4^ cells/insert). After 3 to 5 days, the rat BMECs were seeded at a density of 1.5 × 10^5^ cells/insert onto the opposite side of the inserts to produce rat BMEC monolayers. The *in vitro* BBB cell model was formed 3 to 4 days. Trans-epithelial electrical resistance (TEER) measurements were performed everyday after the co-cultures formed until the TEER value increased to 300 Ω/cm^2^ (Yang et al., 2014) (**Figure 2C**).

#### Transport Experiment in the *In Vitro* BBB Cell Model

The transport experiments were performed by adding 400 μl HBSS containing the compounds to the apical side (AP), with 600μl HBSS to the basolateral side (BL) at the same time. Samples (60 μl) were collected from the BL chamber at the time points of 15, 30, 60, 90, 120, and 180 min, and the corresponding volume of HBSS buffer was immediately added into the BL chamber. The samples were precipitated by methanol, and the concentrations of the compounds in the samples were determined by high-performance liquid chromatography (HPLC). The HPLC conditions were as follows: Agilent TC-C_18_ column (250 × 4.6 mm; 5 μm), methanol 1% ethanoic acid mobile phase (from 10:90 to 90:10, v/v), with gradient elution at a flow rate of 1.0 ml/min. The detection wavelength was 275 nm for these compounds. The run time was 20 min. The apparent permeability coefficients (*P*_app_) were calculated from the following formula: *P*_app_ = (∆Q/∆t)/(*A* × *C*_0_), where ∆Q/∆t is the linear appearance rate of the compound on the BL side (μmol/s), *A* is the surface area of the cell monolayer (cm^2^), and *C*_0_ is the initial concentration of the drug on the AP side (μmol/L).

### Anti-Glioma Effects of Apocynin Derivatives by MTT Assay

The anti-glioma effects of these compounds were evaluated by MTT assay in rat C6 glioma cells, human glioma U87 and U251 cells, and BMECs cells. Brieﬂy, cells were seeded at the density of 4,000 cells/well in 96-well plates overnight. For the first screening, the cells were incubated with 10, 20, 100 μM compounds for 48 h. Then cell viabilities were detected by MTT assay as described above. For the second screening, the cells were incubated with 6.25-, 12.5-, 25-, 50-, and 100μM compounds for 48 and 96 h, and then detected by MTT assay. The OD of each well was measured at 490 nm on an ELISA microplate reader. The inhibitory rates and IC_50_ values were calculated.

### Establishment of Rat C6 Glioma Model and Candidate Drugs Treatment

C6 glioma model was established as previously reported (Xu etal., 2016). The C6 glioma cells in the logarithmic growth phase were collected for implantation. Male Wistar rats (male, 160–180g) were anesthetized with 10% chloral hydrate, and then a hole (1 mm diameter) was drilled in the cranial bone. C6 cells in PBS were injected slowly (3 × 10^6^ cells/rat).

Tumors in the brains of the rats were detected by Bruker 7.0 T Micro-MRI using a T2W RARE sequence (TR/TE 3000/15 ms; slice thickness, 1.0 mm; flip angle, 180°; Matrix 256×256; slice gap, 1.0 mm; FOV, 33×33 mm; time, 4.8 min) 7 days after implantation. Tumor volumes (*V*) were calculated by the following formula: *V* = (4/3 × π × *LHW*)/8, where *L* is the maximum anteroposterior diameter, *H* and *W* are the height and width. Then the rats were sacrificed, and the brain tissues were fixed with formalin, embedded in paraffin, sectioned, and subjected to hematoxylin/eosin (HE) staining and immunohistochemical staining for glial fibrillary acidic protein (GFAP) as previously described (Xu et al., 2016). The sections from each animal were analyzed by a pathologist. Only rats with maximum intracerebral tumor diameter of >5.0 mm were used.

After the successful implantation, the rats were randomly divided into five groups and treated with different drugs by intraperitoneal injection daily: control group (PBS, *n* = 6), TMZ group (25 mg/kg, *n* = 6), D14 group (25 mg/kg, *n* = 6), and D31 group (25 mg/kg, *n* = 6). The body weights were recorded everyday after administration. Thirteen days after treatment, brain tumors in the rats were detected by Bruker 7.0 T Micro-MRI as described above. Tumor volumes were calculated and recorded as above.

### NOX Activity Determination

The effects of candidate drug on NOX activities were measured using cytochrome c reductase (NADPH) Assay Kit (Sigma-Aldrich, St. Louis, MO, USA). Brieﬂy, after the compound treatment (20, 50 μM), the cells were collected and homogenized in an isotonic buffer (pH .5), and centrifuged at 1,000 *g* to obtain the post-nuclear supernatants. Then 50μl samples were added to 950μl working solution (at 25°C) in a 1ml cuvette and mixed by inversion. Then 20μl cytochrome c oxidase inhibitor solution was added. After reaction with 100 μl NADPH solution, the cytochrome c absorbance was monitored at 550 nm by a spectrophotometer.

### Determination of the Reactive Oxygen Species Levels

Intracellular reactive oxygen species (ROS) levels were detected using Reactive Oxygen Species Assay Kit (Jiancheng Bioengineering, Nanjing, China) as per the manufacturer’s instruction. Brieﬂy, after treatment with indicated concentrations of test compound (20, 50 μM) for 48 h, the cells were incubated in 1 ml 2,7-dichloroﬂuorescin diacetate working solution (100 μM) for 30 min at 37°C in the dark, and washed with PBS. Then ﬂuorescence intensities of intracellular DCF were detected using a FACS Calibur ﬂow cytometer (Becton-Dickinson, San Jose, CA, USA). H_2_O_2_ treatment group was used as the positive control group.

### Apoptosis Analysis by Annexin V-FITC/PI Staining

The effects of test compound on apoptosis were detected by Annexin V-FITC/PI staining. Briefly, after treatment with indicated concentrations of test compounds (20, 50 μM) for 48 h, the cells were collected and resuspended in 500-μl detection buffer. Then 5μl PI and 5μl Annexin V-FITC were added to the detection buffer. Then, cells were incubated for 15 min in the dark and analyzed using Flow cytometer (Becton Dickinson) and WinMDI 2.8 software (Scripps Institute, La Jolla, CA, USA).

### Western Blotting Analysis for Apoptosis-Related Proteins

Briefly, after treatment with indicated concentrations of test compound (20, 50 μM) for 48 h, the cells were collected, and total cell lysates and nucleus fractions were extracted. Twenty micrograms of cellular protein were subjected to electrophoresis on 12% SDS-polyacrylamide gels and then transferred to polyvinylidene difluoride membranes. The membranes were first incubated with 5% BSA for 2 h at 37°C and then incubated with the first antibodies overnight at 4°C. After HRP-labeled secondary antibodies (1:1,000-dilution) incubation, protein bands were visualized with the enhanced chemiluminescence (ECL) detection solution. The intensity of bands was quantified using ImageJ 1.43 software (National Institutes of Health, Bethesda, MD, USA).

## Results

### Synthesize and Structure Determination of Apocynin Derivatives

As shown in **Scheme 1**, the key intermediate D4 was synthesized from commercially available D1 by two steps: D1 and D2 reacted for 4 h at 40°C in the presence of K_2_CO_3_ and DMF to produce D3. Successive bromo substitution reactions of D3 with Br_2_ in CHCl_3_ gave the intermediate D4. Treatment of D4 with aniline or para-fluoroaniline in the presence of NaHCO_3_ and CH_3_CN yielded the final products D5 and D6.

Another key intermediate D11 was yielded by bromo substitution reactions of D1 with CuBr_2_ in ethyl acetate. Treatment of D11 with aniline, para-fluoroaniline, 2,4-dimethoxyaniline, 2,4-difluoroaniline, or 4-chloroaniline in the presence of NaHCO_3_ and DMF yielded the final products D13, D14, D16, D21, and D22.

Treatment of D11 with benzyl mercaptan 2-chlorobenzyl mercaptan or 4-fluorobenzyl mercaptan in the presence of triethylamine and dichloromethane yielded the final products D31, D33, and D34.

All the novel apocynin derivatives are fully characterized by means of ^1^H NMR, ^13^C NMR, and HRMS measurements, and their structures are shown in **Figure 1**. NMR determinations showed purities >98% for all compounds.

### *In Silico* ADMET Prediction of the Apocynin Derivatives

As shown in **Table 1**, all compounds possessed good absorption compared with compound D1, whereas all compounds possessed much higher hydrophobicity and poorer solubility in aqueous media compared with compound D1. Compound D5, D6, D31, D33, and D34 were predicted to be high BBB penetrant, while D13, D14, D21, and D22 were predicted to be medium BBB penetrant. Only D16 was predicted to have low BBB penetrant level. All compounds were predicted to possess highly bound (≥90% bound) to plasma proteins except for compound D1. Compounds D5, D6, D14, D22, D31, D33, and D34 were predicted to be CYP2D6 inhibitors. And compounds D14, D16, D21, D22, and D34 were predicted to exhibit hepatotoxicity. Further biological experiments are required to obtain additional data for testing these predictions.

**Table 1:** Predicted pharmacokinetic properties of Apocynin derivatives.

Compound	Absorption Level	AlogP98	BBB Level	CYP2D6	Hepatotoxicity	PPB	PSA2D	Solubility Level
D1	0	1.311	2	FALSE	FALSE	FALSE	47.046	4
D5	0	4.445	1	TRUE	FALSE	TRUE	47.971	2
D6	0	4.65	1	TRUE	FALSE	TRUE	47.971	2
D13	0	2.636	2	FALSE	FALSE	TRUE	59.856	3
D14	0	2.841	2	TRUE	TRUE	TRUE	59.856	3
D16	0	2.603	3	FALSE	TRUE	TRUE	77.716	3
D21	0	3.047	2	FALSE	TRUE	TRUE	59.856	3
D22	0	3.3	2	TRUE	TRUE	TRUE	59.856	3
D31	0	4.04	1	TRUE	FALSE	TRUE	47.046	2
D33	0	3.376	1	TRUE	FALSE	TRUE	47.046	3
D34	0	3.581	1	TRUE	TRUE	TRUE	47.046	3

AlogP98: lipophilicity descriptor. Absorption Level: 0, good; 1, moderate; 2, low; 3, very low. BBB level: 0, very high penetrate; 1, high; 2, medium; 3, low; 4, undefined. CYP2D6 prediction: to evaluate the CYP2D6 inhibitory effects of drugs using the cutoff Bayesian score of 0.161 (obtained by minimizing the total number of false positives and false negatives). Hepatotoxic prediction: to evaluate the potential human hepatotoxicity using the cutoff Bayesian score of −4.154 (obtained by minimizing the total number of false positives and false negatives). PPB prediction: to devaluate the plasma proteins bound effects of drugs (highly bound: ≥90% bound, using the cutoff Bayesian score of −2.209 obtained by minimizing the total number of false positives and false negatives). PSA-2D: Polar surface area. Solubility level: 0, extremely low; 1, very low; 2, low; 3, good; 4, optimal; 5, too soluble. PPB, plasmatic protein binding.

### BBB Penetrant Assay of Apocynin Derivatives In Vitro

After isolation of BMECs cells, the cytotoxicity of these compounds on rat BMECs was evaluated. As shown in **Figure 2B**, compared with the control group, 100-μM compounds D1, D13, D14, D16, D31, D33, D34, and TMZ could significantly inhibit the cell viability of rat BMECs (*P* < 0.05). There were no obvious effects for 20 μM of all compounds on BMECs. Based on the inhibitory effects of these compounds to rat BMECs, the safely tested concentrations of these compounds were chosen for the BBB penetrate assay.

The BBB cell model was established by co-culture of rat BMECs and astrocytes in transwell polycarbonate filters, and the TEER was measured everyday. Only filters with TEER value > 300 Ω/cm^2^ were used for the BBB penetrate assay. The *P*_app_ values for these compounds across the BBB cell layer were summarized in **Table 2**. The *P*_app_ value of TMZ was 7.74 × 10^−6^ cm/s. D5, D6, D31, D33, and D34 had much higher permeation than D1 and TMZ, and the *P*_app_ values of D5, D6, D31, D33, and D34 were 10.34 ± 0.31, 8.68 ± 0.45, 11.97 ± 0.94, 9.58 ± 2.35, and 11.89 ± 1.53 × 10^−6^ cm/s, respectively. D16 had the lowest permeation across the BBB cell layer, with the *P*_app_ values of 2.54 ± 0.58 × 10^−6^ cm/s, respectively.

**Table 2:** Apparent permeability coefficients (P_app_) of the compounds in BBB cell model.

Compound	Standard curve	C_0_ (μM)	*P*_app_ × 10^−6^ (cm/s)
D1	*y* = 85.9121x + 6.9052	20	4.95 ± 0.39
D5	*y* = 47.5216x + 2.4375	100	10.34 ± 0.31
D6	*y* = 37.0426x + 3.9065	100	8.68 ± 0.45
D13	*y* = 121.2051x −1.4975	20	5.41 ± 0.52
D14	*y* = 224.1600x + 10.6510	20	4.24 ± 1.30
D16	*y* = 110.5854x + 2.5641	20	2.54 ± 0.58
D21	*y* = 124.1046x + 5.4653	100	6.08 ± 0.65
D22	*y* = 109.6889x + 2.8041	100	5.42 ± 0.58
D31	*y* = 107.0040x + 9.0208	20	11.97 ± 0.94
D33	*y* = 111.0032x + 2.7632	20	9.58 ± 2.35
D34	*y* = 173.8767x −14.2328	20	11.89 ± 1.53
TMZ	*y* = 134.6431x + 3.4643	20	7.74 ± 0.34

*C*_0_: the initial concentrations of compounds. P_app_: Transport of the compounds from the apical to basolateral side at 90 min. Values were the means ± SD (n = 3-4).

TMZ, temozolomide.

### Anti-Glioma Effects of Apocynin Derivatives on Different Glioma Cells

**Figure 3:**
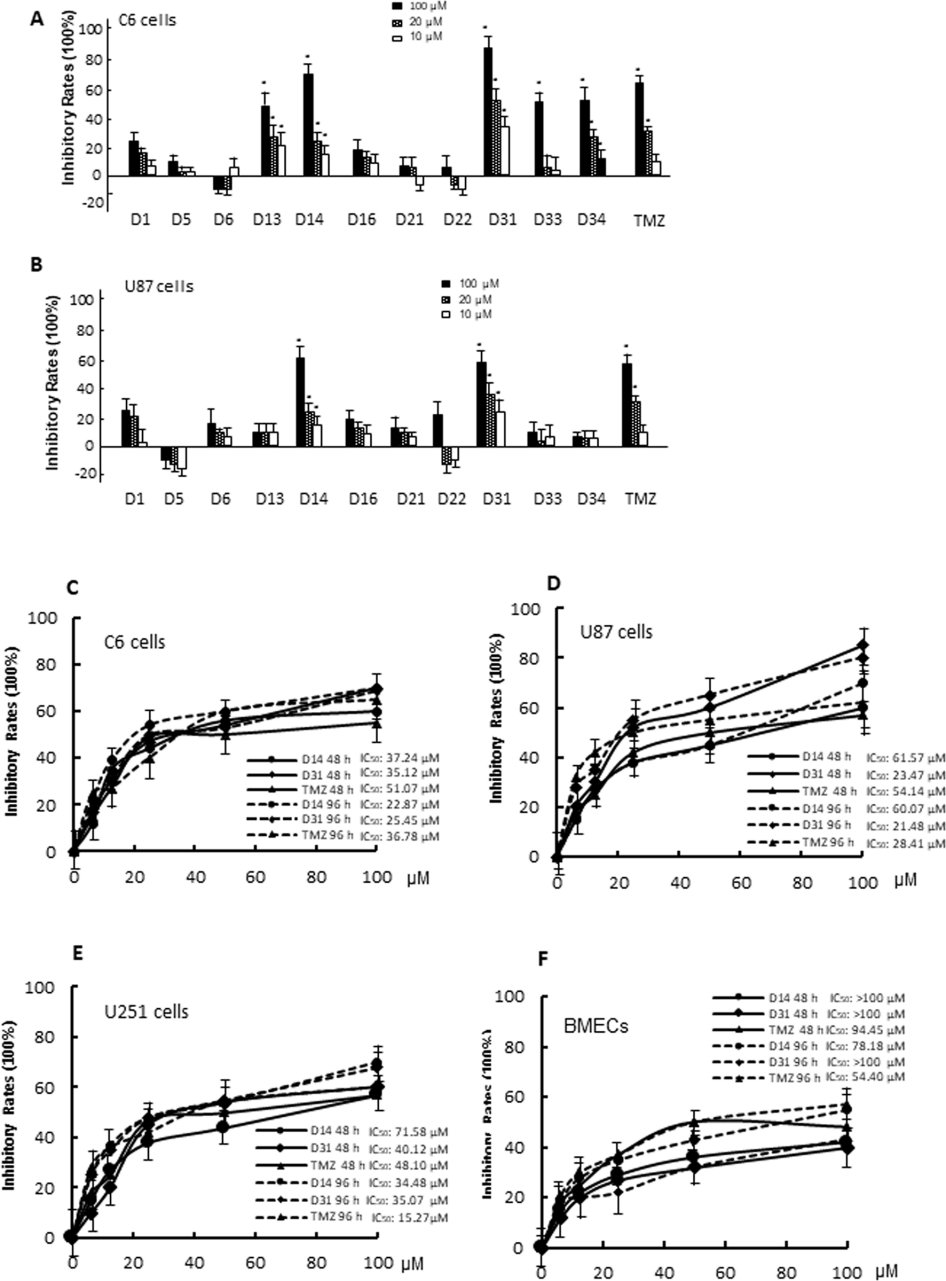
The cytotoxicity of apocynin derivatives on C6 and U87 glioma cells. **(A**, **B)**, Inhibitory effects of 10, 20, 100 μM of apocynin derivatives on viability of C6 and U87 cells. *P< 0.05 compared with D1 in the same concentration. **(C**, **D**, **E**, **F)**, Inhibitory curves of D14 and D31 on viabilities of C6 cells, U87 cells, U251 cells, and BMECs at the time points of 48 and 96 h.

*In vitro* anti-glioma effects of the compounds were evaluated by MTT assay. For the first screening, we evaluated 10-, 20-, and 100μM apocynin derivatives on proliferation of C6 and U87 cells. As shown in **Figures 3A, B**, different compounds exhibit different effects on the cells. Compared with D1, D13, D14, D31, D33, D34, TMZ showed significant anti-proliferation effects on C6 cells in dose-dependent manners (*P* < 0.05). However, only D14, D31, and TMZ showed significant anti-proliferation effects on U87 cells (*P* < 0.05). Thus, the anti-proliferation effects of D14 and D31 on different glioma cell lines and normal BMECs were evaluated in five different concentrations for the second screening, and the inhibitory curves and IC_50_ values are shown in **Figures 3C–F**. It can be found that D14 and D31 showed significant anti-proliferation effects in dose- and time-dependent manners. Also, compared with normal cells, D14 and D31 exhibited more potent inhibitory effects on tumor cells.

### D31 Inhibited Tumor Growth Without Affecting the Body Weights of Glioma-Bearing Rats

**Figure 4:**
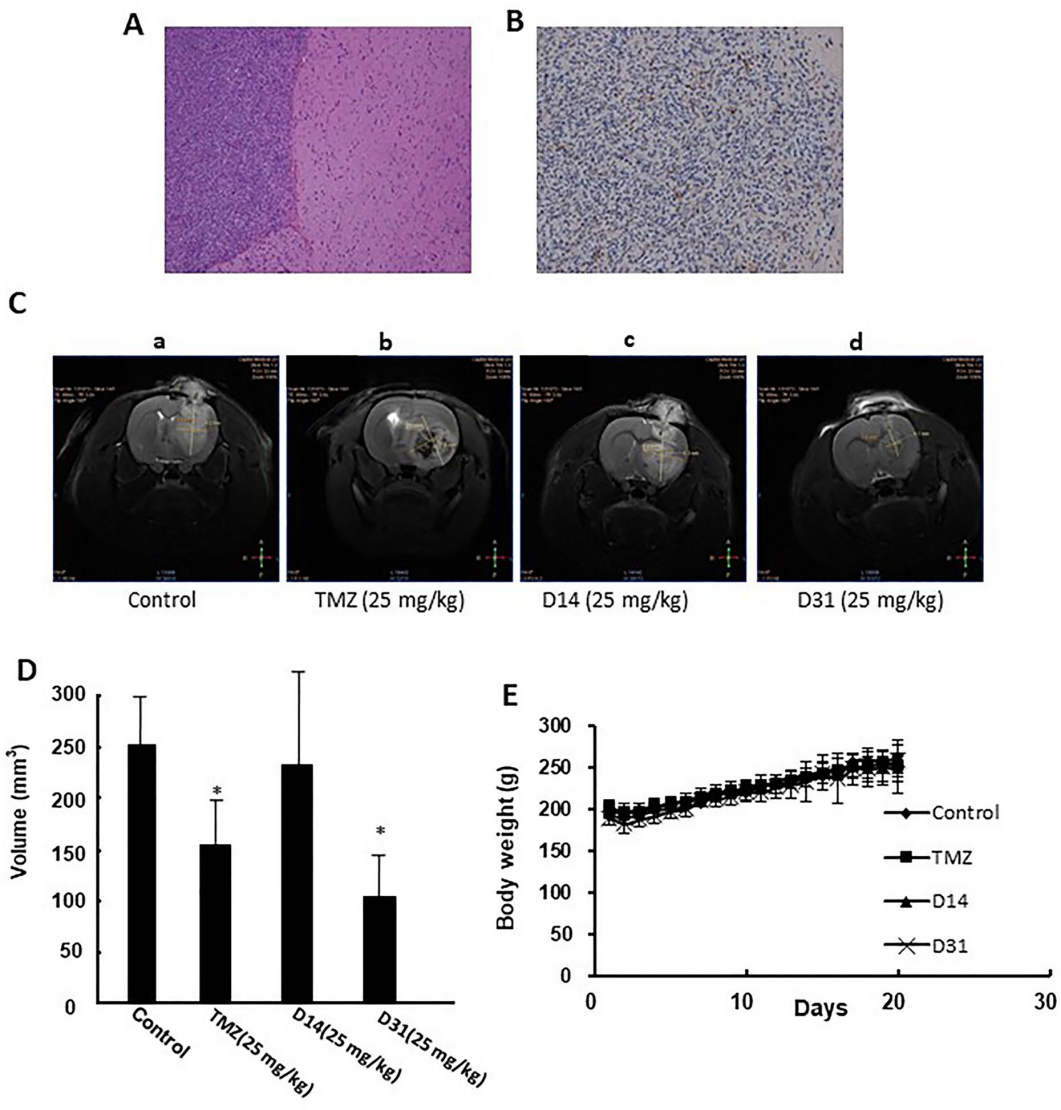
Anti-glioma effects of D14 and D31 on C6 glioma model in rats. **(A)**, Representative HE staining result of the implanted gliomas (×100); **(B)** Representative immunohistochemical staining for GFAP in the gliomas tissues (×100); **(C)** Representative MRI images of brains of glioma-bearing rats with or without drugs treatment. **(D)** Tumor volumes of glioma-bearing rats with or without drugs treatment. The results are expressed as mean values ± SD. *P < 0.05, compared to the control group. **(E)** Body weight changes of Wistar rats before and after drugs treatment.

The rat C6 glioma model was established and confirmed by MRI, HE, and immunohistochemical staining. Obvious pathologic changes can be found in tumor tissue areas as shown in HE staining result (**Figure 4A**). Positive GFAP immunohistochemical staining can be found in the tumor area, confirming that the tumor tissues were astrocytic origin (**Figure 4B**).

Antiglioma effects of D14 and D31 were then evaluated in the C6 glioma-bearing rats. As shown in **Figures 4C, D**, tumor volumes in the D31- and TMZ-treated groups were significantly decreased compared to the control group (*P* < 0.05). Moreover, D14 treatment showed no obvious growth inhibitory effects on C6 glioma (*P* > 0.05). Also, compared with the control group, no obvious body weight changes were found in D14-, D31-, and TMZ-treated groups (*P* > 0.05, **Figure 4E**). These results suggested that D31 treatment could significantly decrease tumor growth without affecting the body weights of the glioma-bearing rats.

### D31 Induced ROS Generation

**Figure 5:**
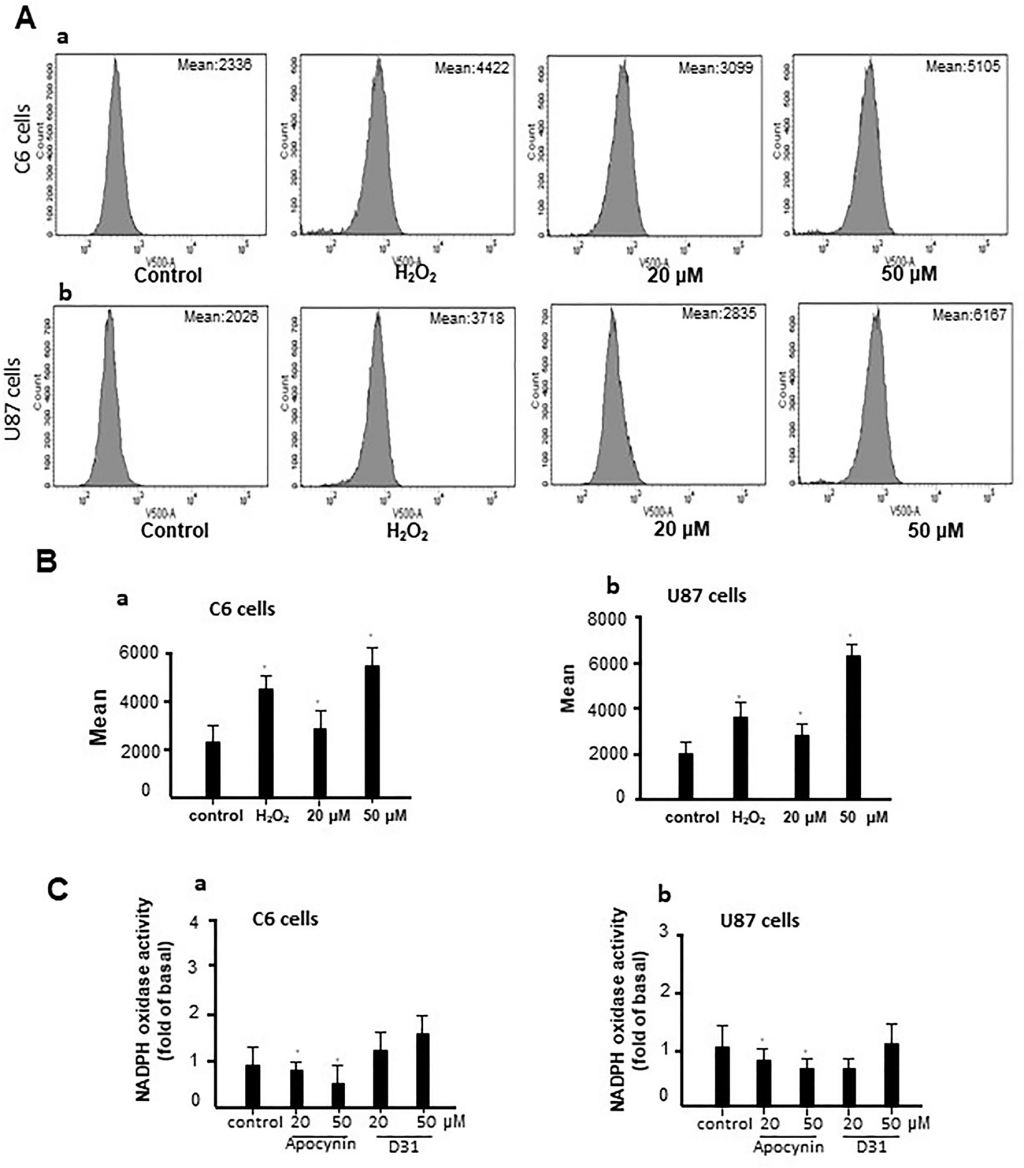
D31 induced ROS generation without affecting NADPH oxidase activity. **(A)**, Effects of D31 on ROS production analyzed by flow cytometry in C6 (a) and U87 (b) cells; **(B)**, Mean fluorescent intensity of C6 (a) and U87 (b) cells after D31 treatment; **(C)**, Effects of apocynin and D31 on NADPH oxidase activities in C6 (a) and U87 (b) cells. The results are expressed as mean values ± SD. *P< 0.05 compared with control.

To investigate the mechanisms involved in the anti-glioma effects of D31, we examined the generation of ROS in the cells. As shown in **Figures 5A, B**, D31 induced significant increases in the fluorescence intensity of DCF in both C6 and U87 cells compared with the control groups (*P* < 0.05).

Since apocynin was proved to be a NOX inhibitor, we also detected the effects of D31 on NOX activities. As shown in **Figure 5C**, apocynin significantly inhibited NOX activities in both C6 and U87 cells (*P* < 0.05), whereas D31 showed no obvious effects on NOX activities in both C6 and U87 cells compared with the control groups (*P* > 0.05). The above result indicated that D31 induced ROS generation without affecting NOX activities.

### D31 Induced Cell Apoptosis and Inhibited NF-κB Activation

**Figure 6:**
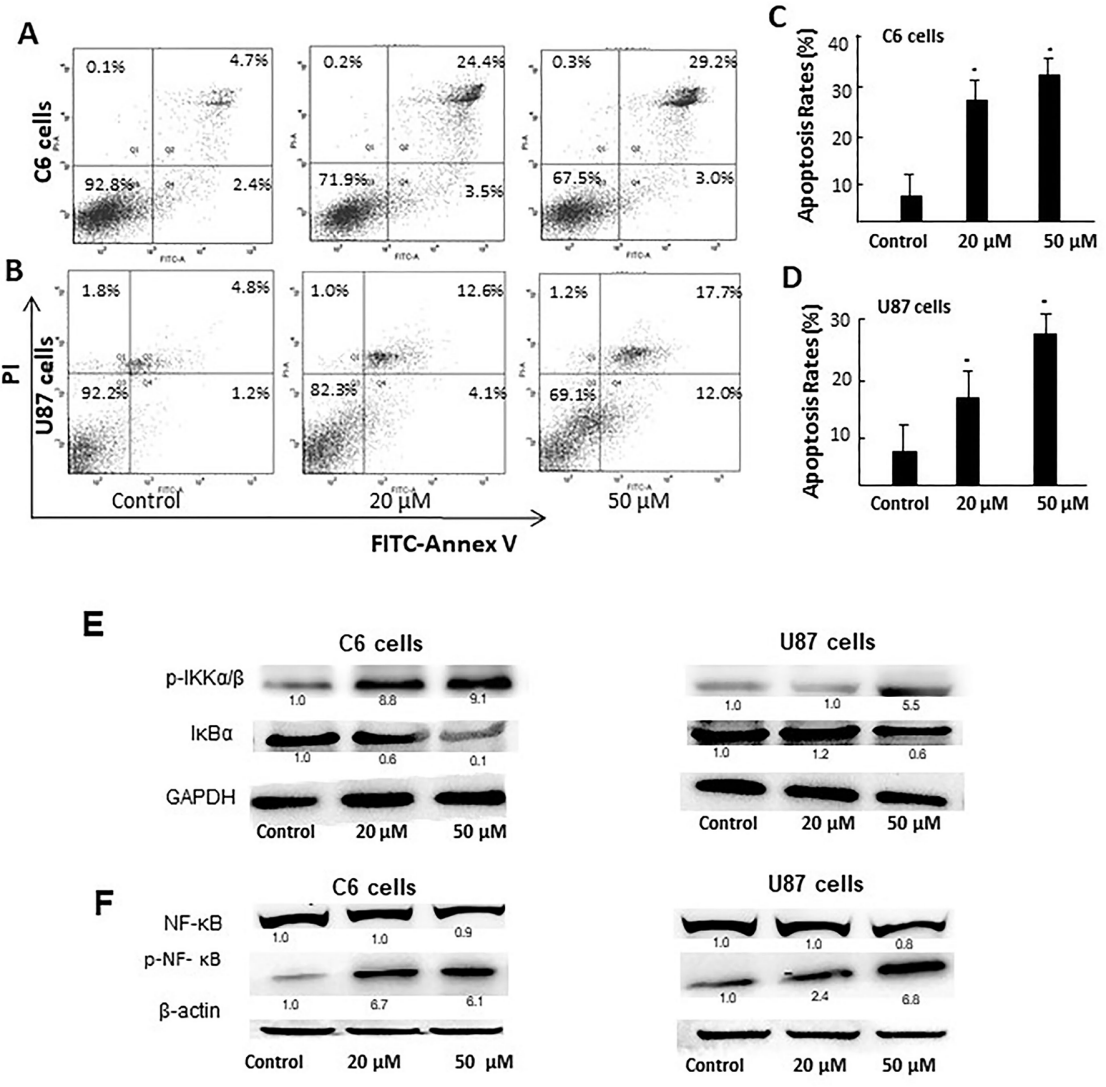
D31 induced apoptosis of cancer cells and inhibited NF-κB activation. **(A**, **B)**, Detection of the apoptosis of C6 and U87 cells with or without D31 treatment (20 and 50 μM) by FITC-Annexin V/PI double staining. **(C**, **D)**, the quantity of apoptosis rates for C6 and U87 cells. The results are expressed as mean values ± SD. *P< 0.05 compared with control. **(E)**, Western blot analysis for p-IKKα/β and IκBα expressions in C6 and U87 cells after treatment with D31 (20 and 50 μM) for 48 h. **(F)**, Western blot analysis for nucleus NF-κB p65 and p-NF-κB p65 expression in C6 and U87 cells after treatment with D31 (20 and 50 μM) for 48 h.

We then carried out Annexin V-FITC/PI staining to detect the effect of D31 on apoptosis of cancer cells. As shown in **Figures 6A–D**, treatment of C6 and U87 cells with D31 significantly increased the cell apoptosis (*P* < 0.05). After treatment with 0-, 20-, and 50-μM D31, the apoptotic cell rates of C6 cells were 7.1 ± 3.3t, 27.9 ± 4.2%, and 32.2 ± 5.0%, and that of the U87 cells were 7.0 ± 2.4%, 16.7 ± 3.1%, and 29.7 ± 4.7%, respectively. Also, more obvious apoptosis inducing effects can be observed in C6 cells. It can also be found that the effects of D31 treatment on the late apoptosis were more obvious than that on the early apoptosis.

Since NF-κB activation was reported to be closely related with both apoptosis and ROS generation, the expressions of NF-κB pathway-related proteins were detected by western blot. As shown in **Figure 6E**, p-IKKα/β expressions were obviously increased, whereas IκBα expressions were obviously decreased after D31 treatment in both cell lines. As shown in **Figure 6F**, nucleus p-NF-κB p65 expressions were obviously increased after D31 treatment in both cell lines. This result indicated that D31 increased p-IKKα/β, and then induced IκBα degradation, NF-κB phosphorylation, and nucleus translocation.

## Discussion

The delivery of anti-glioma drugs across the BBB is considered the major obstacle for their efficacy against glioma (Zhang et al., 2017b). The BBB penetration rate of many drugs can be increased after structure modification (Oldendorf et al., 1972). For example, BBB penetration rate of codeine, 3-hydroxymethylation of morphine, is 10 times higher than that of morphine. BBB penetration rate of heroin, diacetylmorphine, is 100 times higher than that of morphine. Phenobarbital, 5-ethyl-5-phenylbarbituric acid, shows increased BBB penetration rate than that of barbituric acid (Liu et al., 2007).

Apocynin is a natural polyphenolic compound with multiple biological activities and attracts more and more attention for its potential therapeutic applications in central nervous system disease (‘t Hart et al., 2014; Hou et al., 2019). However, the BBB permeation ability of apocynin is quite limited (Wang et al., 2008). The structure modifications of apocynin were seldomly carried out to enhance its BBB penetration rate and explore its potential use in the central nervous system disorders. Considering the bioactivity of apocynin, we designed a series of derivatives based on the skeleton of 3-methoxy-4-hydroxy benzaldehyde. The main modification was focused on the aldehyde group. The substituents included phenylamino unit and benzylthio unit with different groups on the benzene ring. All the synthesized apocynin derivatives were fully characterized by ^1^H NMR, and ^13^C NMR and the structures are shown in **Figure 1**.

Then, we performed *in silico* ADMET prediction of the apocynin derivatives using BIOVIA Discovery Studio 2016 (**Table 1**). For the BBB penetration prediction, compounds D5, D6, D31, D33, and D34 were predicted to have higher BBB penetration levels compared with apocynin. Then, we performed *in vitro* BBB penetration assay for testing this. The result showed that *P*_app_ values of D5, D6, D31, D33, and D34 were higher than that of apocynin (**Table 2**), which consisted the prediction result.

Apocynin was proven to be an efficient NOX inhibitor by many research groups (Min et al., 2018; Du et al., 2019; Hou et al., 2019). NOX family was a major source of ROS production and was regarded to be an important therapeutic target in many diseases (Roy et al., 2015; Ma et al., 2018). NOX family was also found to play important roles in human cancers (Roy et al., 2015; Joo et al., 2016). It was shown that NOX1 supported the proliferations of colon cancer cells by regulating ROS-dependent signal pathways (Juhasz et al., 2017). It was also shown that high expression of NOX1/2/5 was associated with poor prognosis of HCC patients, and NOX4 and DUOX1 expressions correlated with better overall survival of HCC patients (Eun et al., 2017).

Thus, we first evaluated the *in vitro* anti-glioma effects of these derivatives on C6 and U87 cells, and D14 and D31 showed obvious anti-proliferation effects on both C6 and U87 cells. Then, the anti-glioma effects of D14 and D31 were then evaluated in the C6 glioma-bearing rats. The result showed that D31 treatment, but not D14 treatment, significantly inhibited tumor growth without affecting the body weights of the glioma-bearing rats (**Figure 4**). The reason why D14 showed no obvious anti-glioma effects *in vivo* might be related with its relatively low BBB penetration ability. D31 might be developed as an effective anti-glioma agent, and its antiglioma mechanism was further studied.

The present result showed that D31 could significantly induce ROS generation without affecting NOX activities (**Figure 5**), suggesting that D31 induced ROS generation might be related with other potential sources of ROS. Additionally, Annexin V-FITC/PI staining showed that D31 significantly induced apoptosis of C6 and U87 cells dose-dependently (**Figure 6**). NF-κB is a nuclear transcription factor regulating expressions of a large number of genes, and has been shown to play a critical role in ROS induced cell apoptosis (Liu et al., 2014). It was found that apocynin inhibited cancer cell proliferations *via* down-regulating cyclin D1, which might be related with NF-κB activation in rat prostate cancer cell lines (Suzuki et al., 2013). The present result showed that D31 increased p-IKKα/β, induced IκBα degradation, NF-κB phosphorylation, and nucleus translocation. These results indicated that D31 might induce apoptosis by inhibiting NF-κB activations.

Overall, our data demonstrated that D31 inhibited growth and induced apoptosis of glioma, which might be caused by ROS-related NF-κB activation. The current study suggested that D31 could be further explored for its potential use in anti-glioma therapy.

## Data Availability

The raw data supporting the conclusions of this manuscript will be made available by the authors, without undue reservation, to any qualified researcher.

## Ethics Statement

All animal experiment procedures followed the National Institutes of Health guide for the care and use of Laboratory animals (NIH Publications No. 8023, revised 1978) and performed with the approval from the Capital Medical University Ethics Committee in Beijing, China (number 37363).

## Author Contributions

TY wrote the article and performed some experiments. D-WZ, WS, A-CG, and J-PW performed some of the experiments and analyzed the data. Y-JW designed some of the experiments. QW designed most of the experiment and revised the article. All authors read and approved the final manuscript.

## Funding

This work was supported by National Key R&D Program of China (grant 2017YFC1307500); Beijing-Tianjin-Hebei Cooperative Basic Research Program (grant H2018206435); National Natural Science Foundation (grant 81870935); and Center for CNS Drug Discovery.

## Conflict of Interest Statement

The authors declare that the research was conducted in the absence of any commercial or financial relationships that could be construed as a potential conflict of interest.
