# Supplementary figures and images for: Synthesis and Evaluations of Novel Apocynin Derivatives as Anti-Glioma Agents
Source: Front Pharmacol. 2019 Sep 3;10:951. doi: 10.3389/fphar.2019.00951 (PMC6733959; doi:10.3389/fphar.2019.00951)

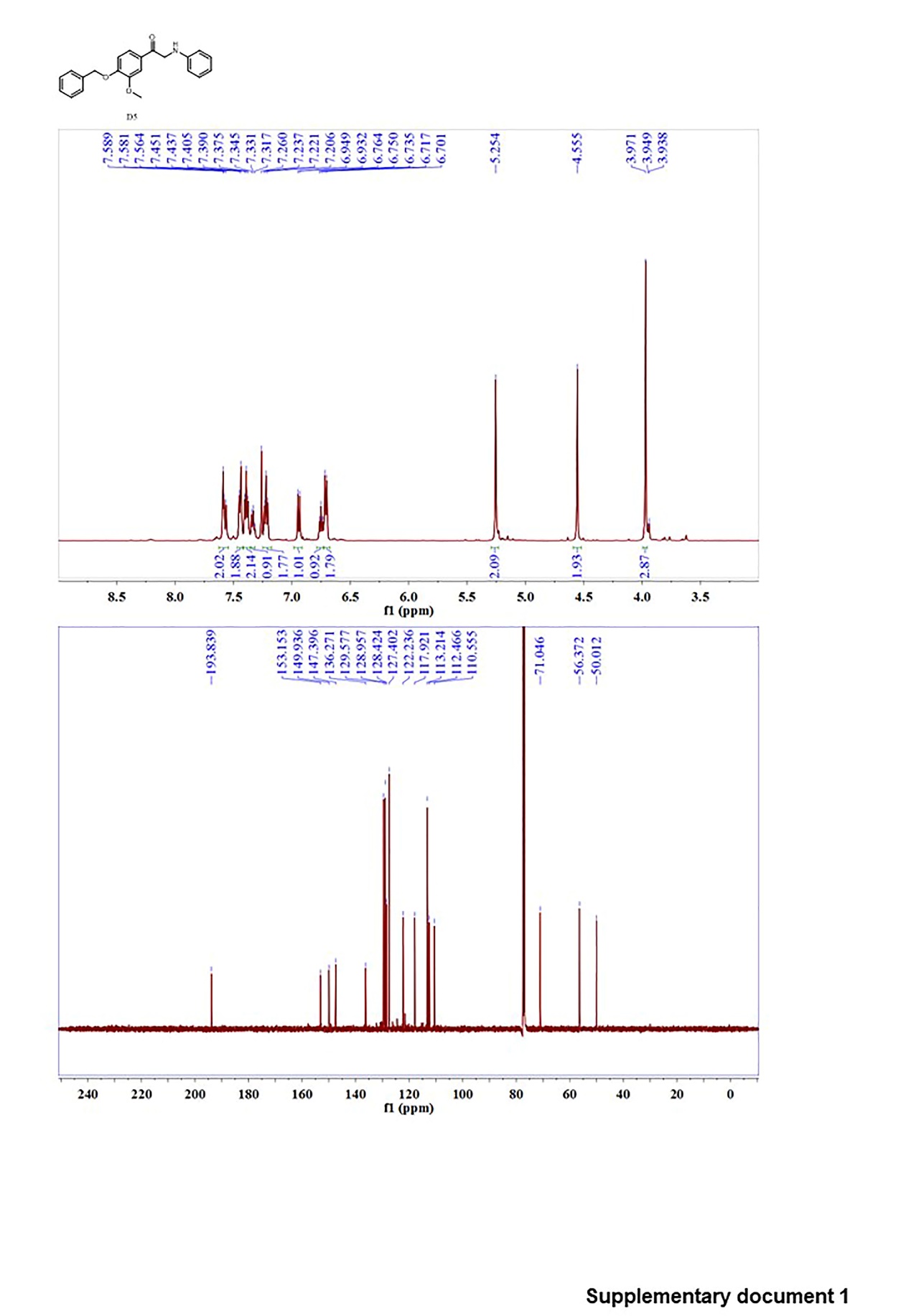


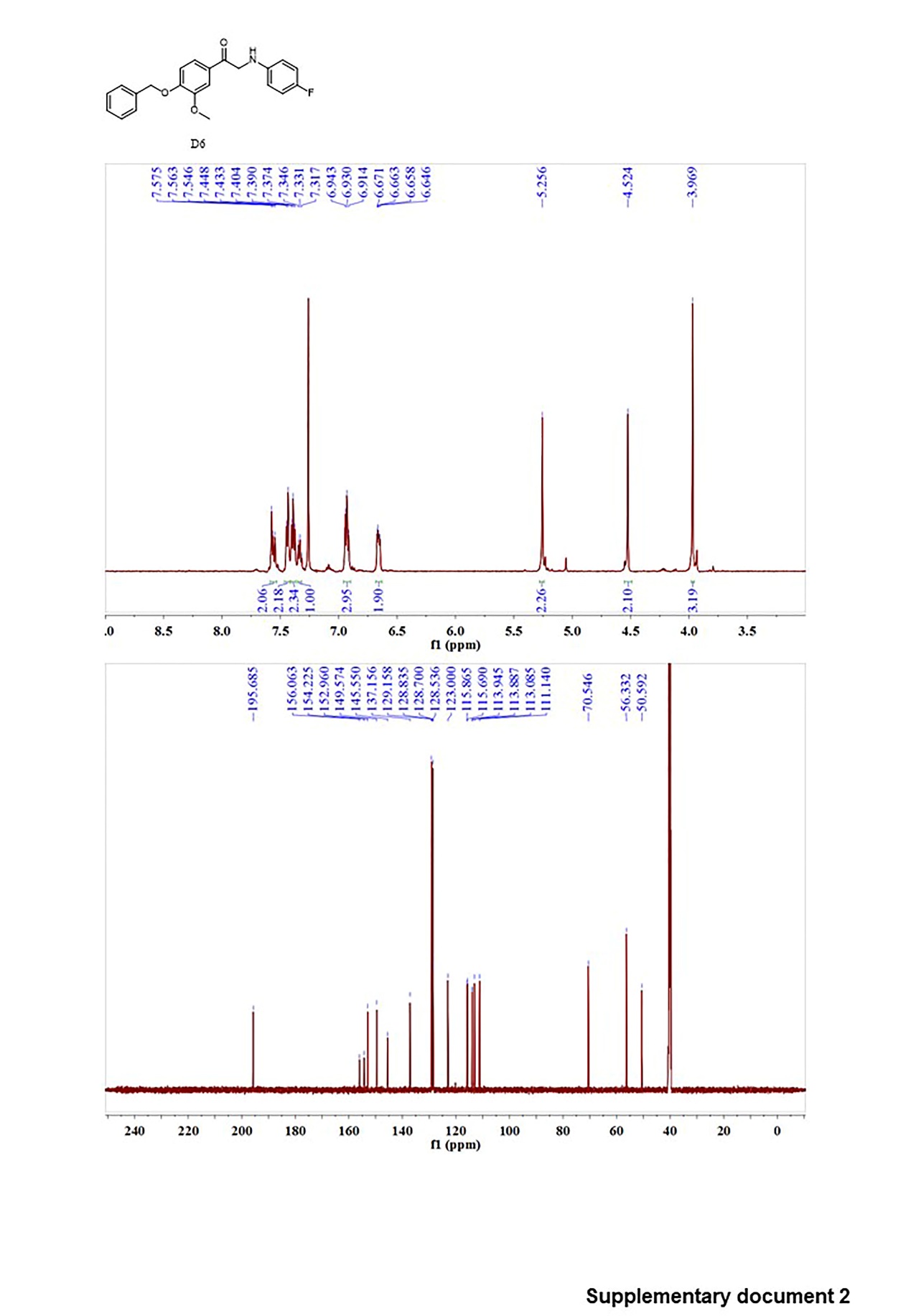


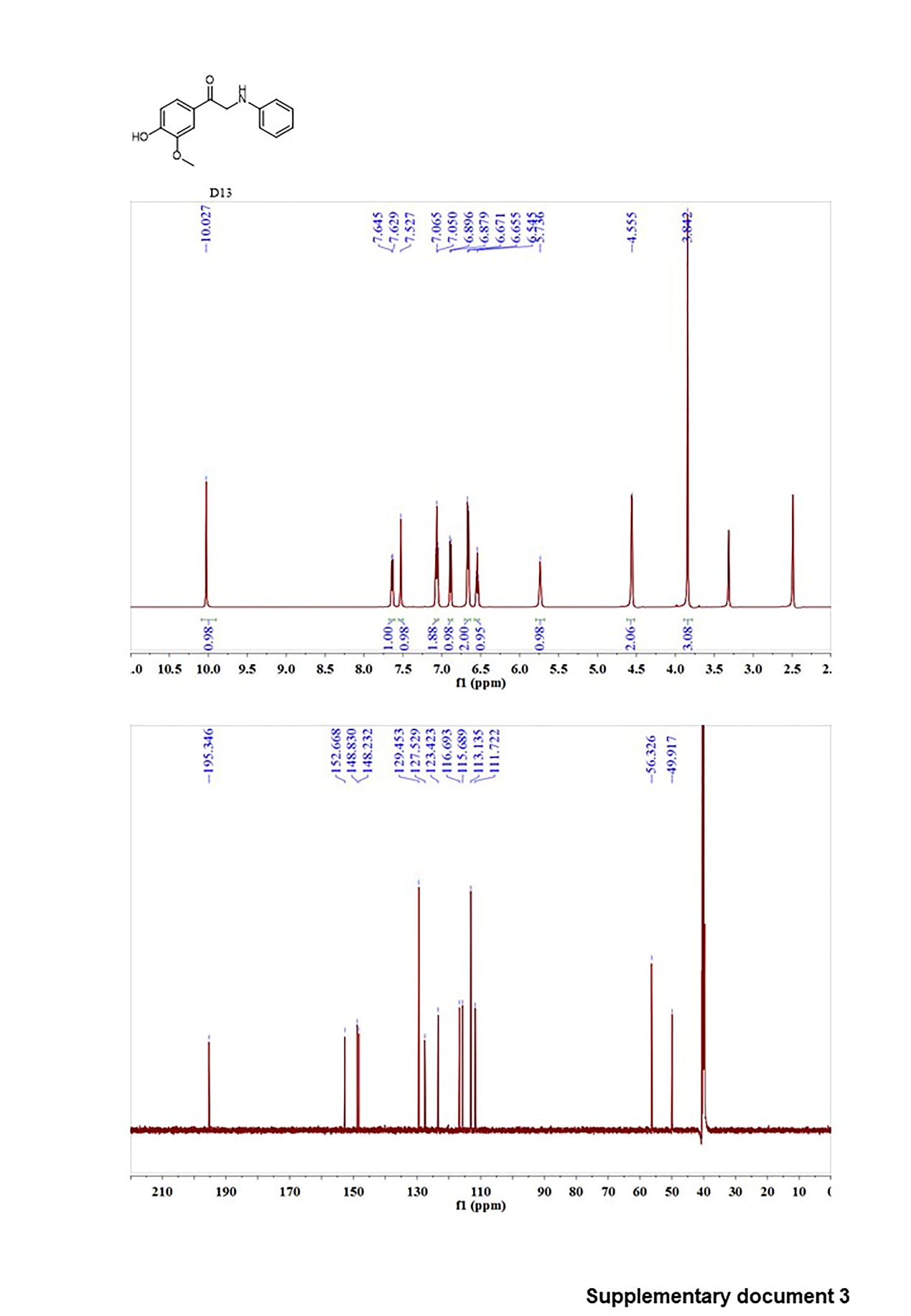


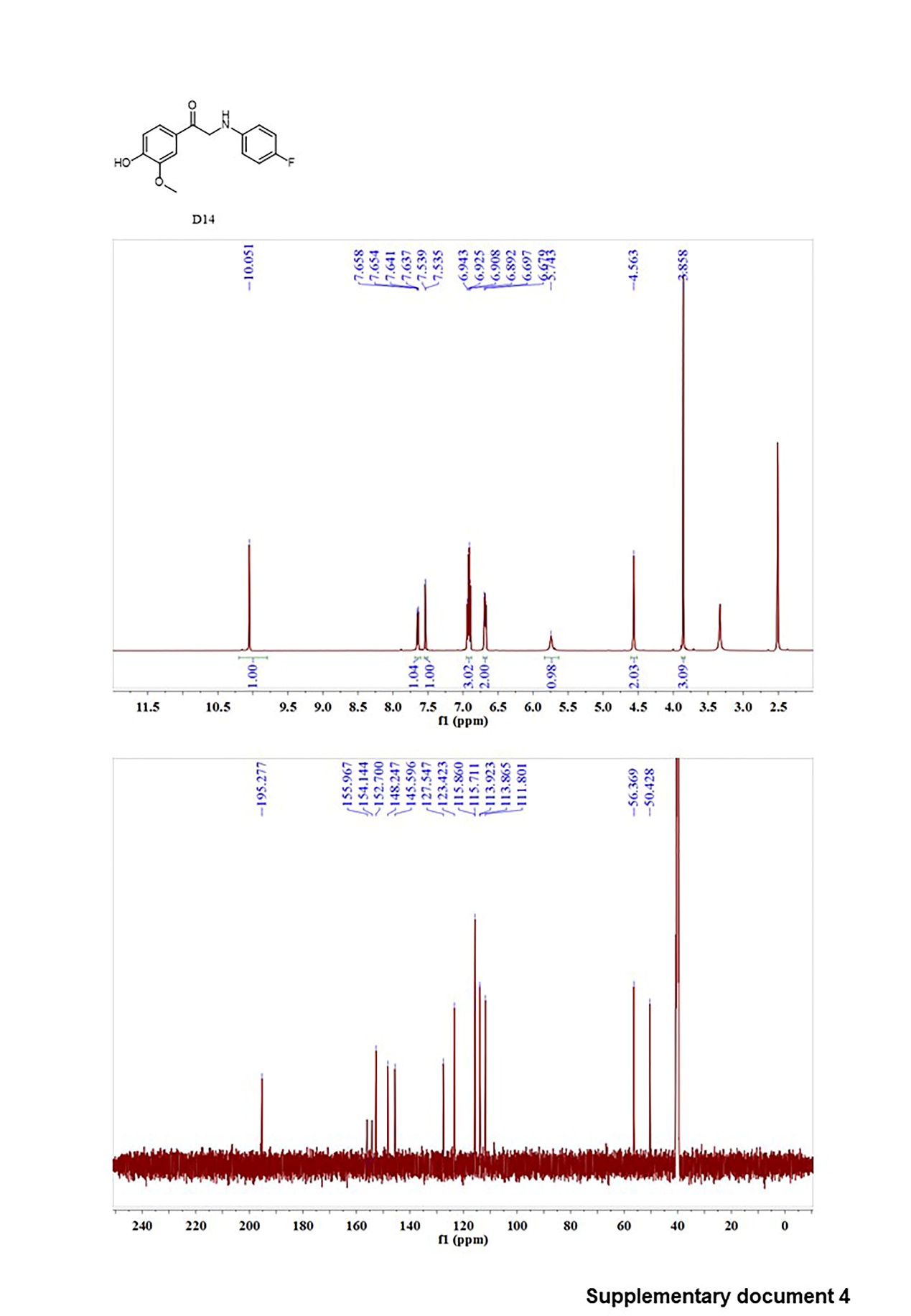


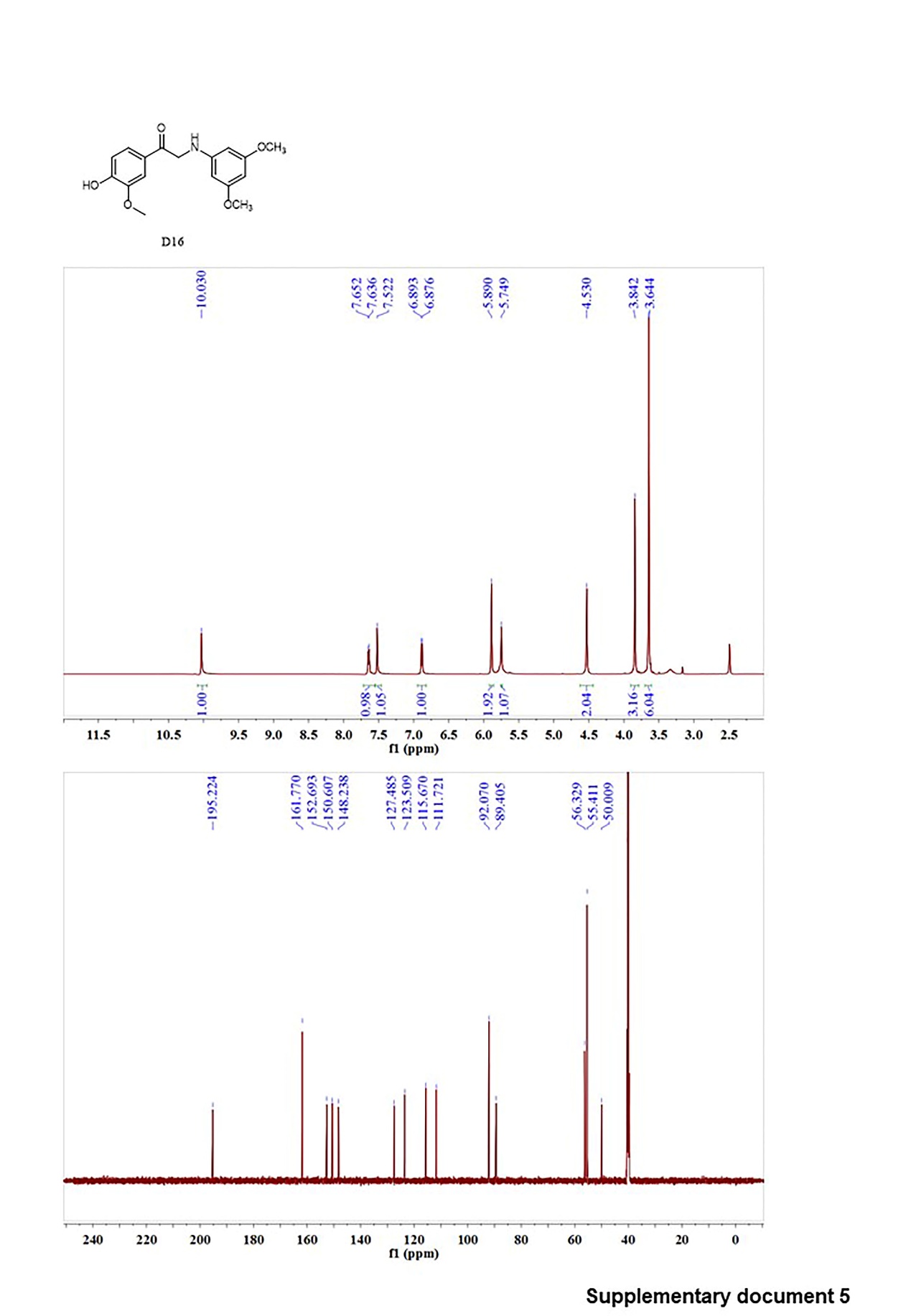


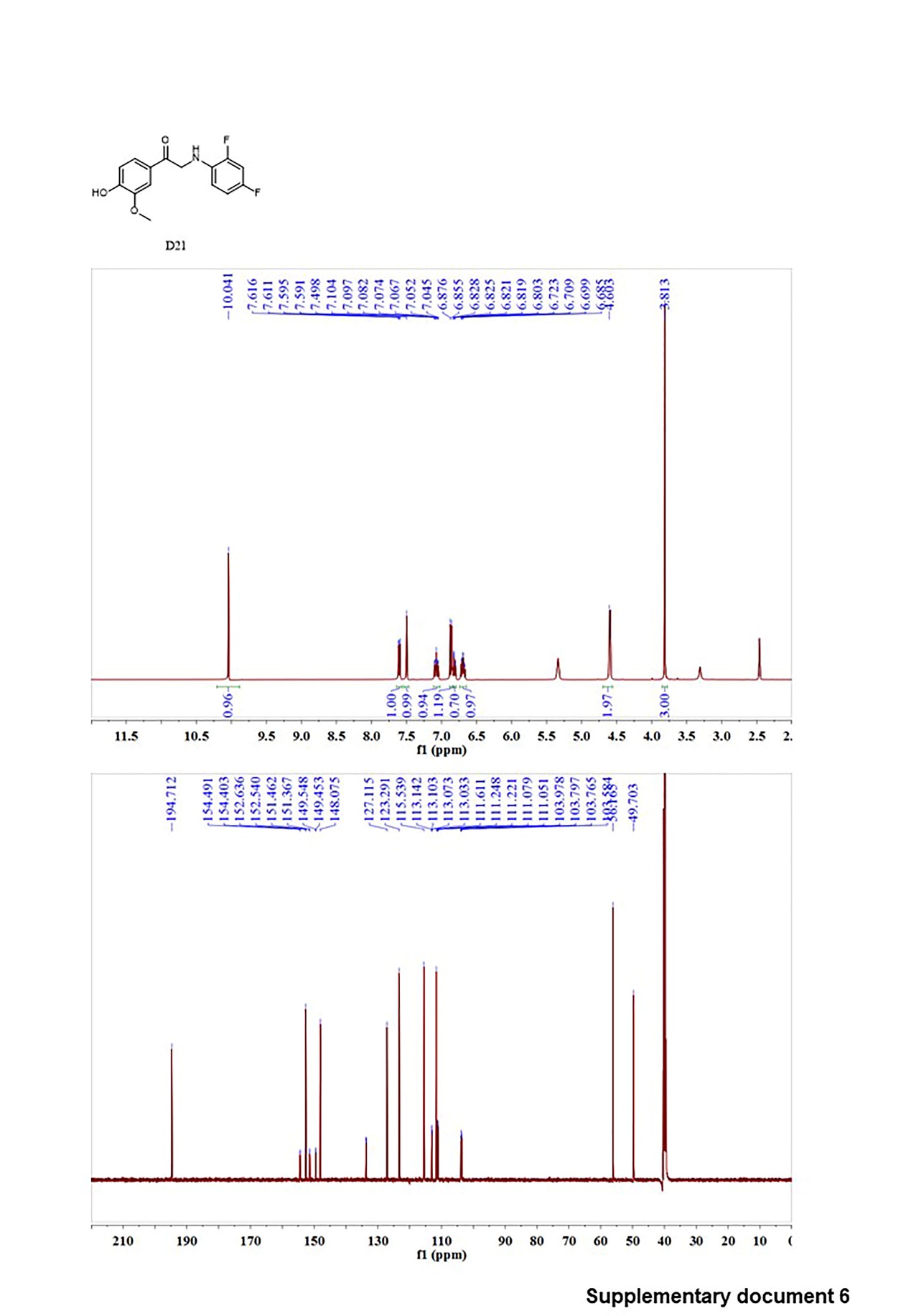


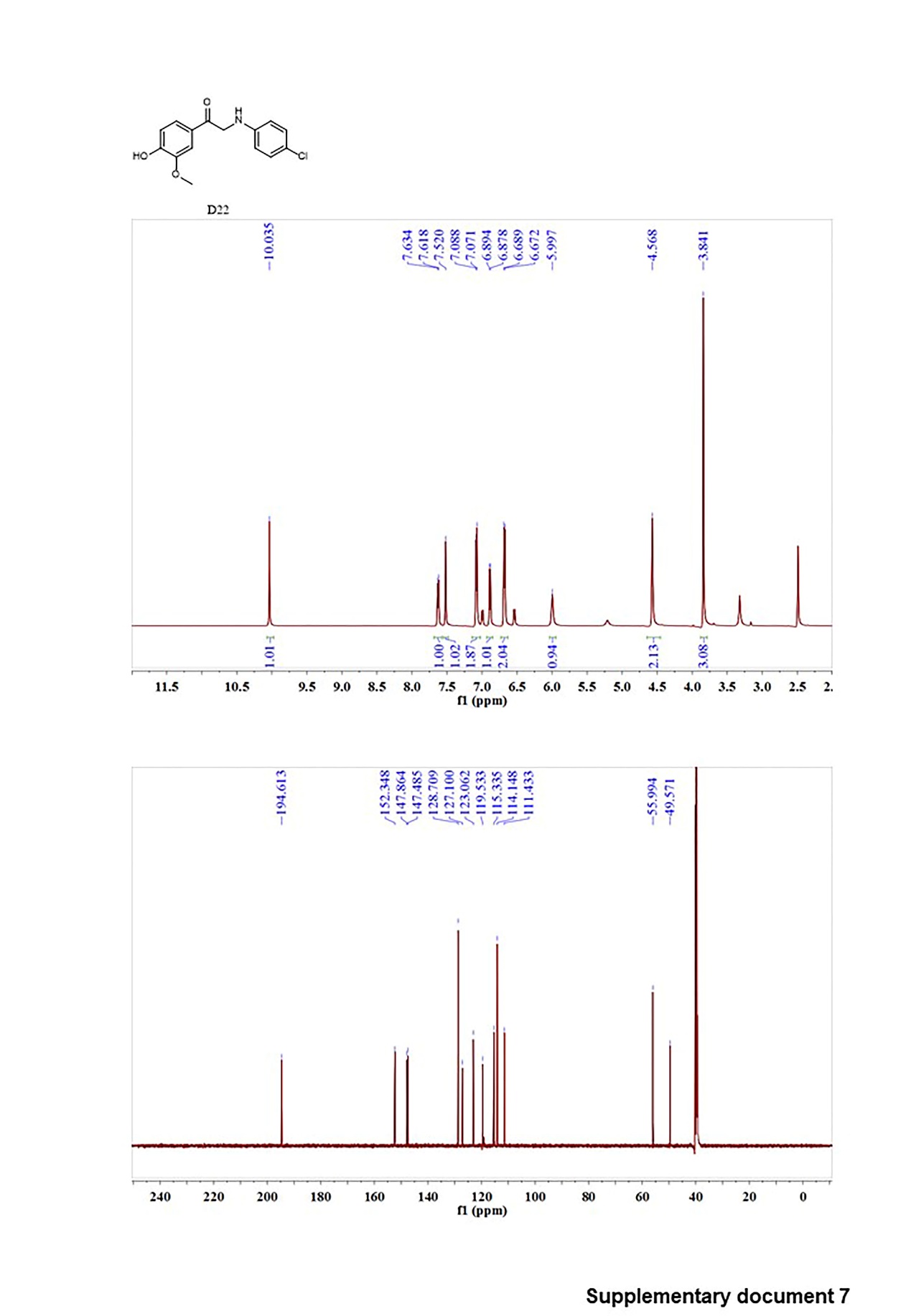


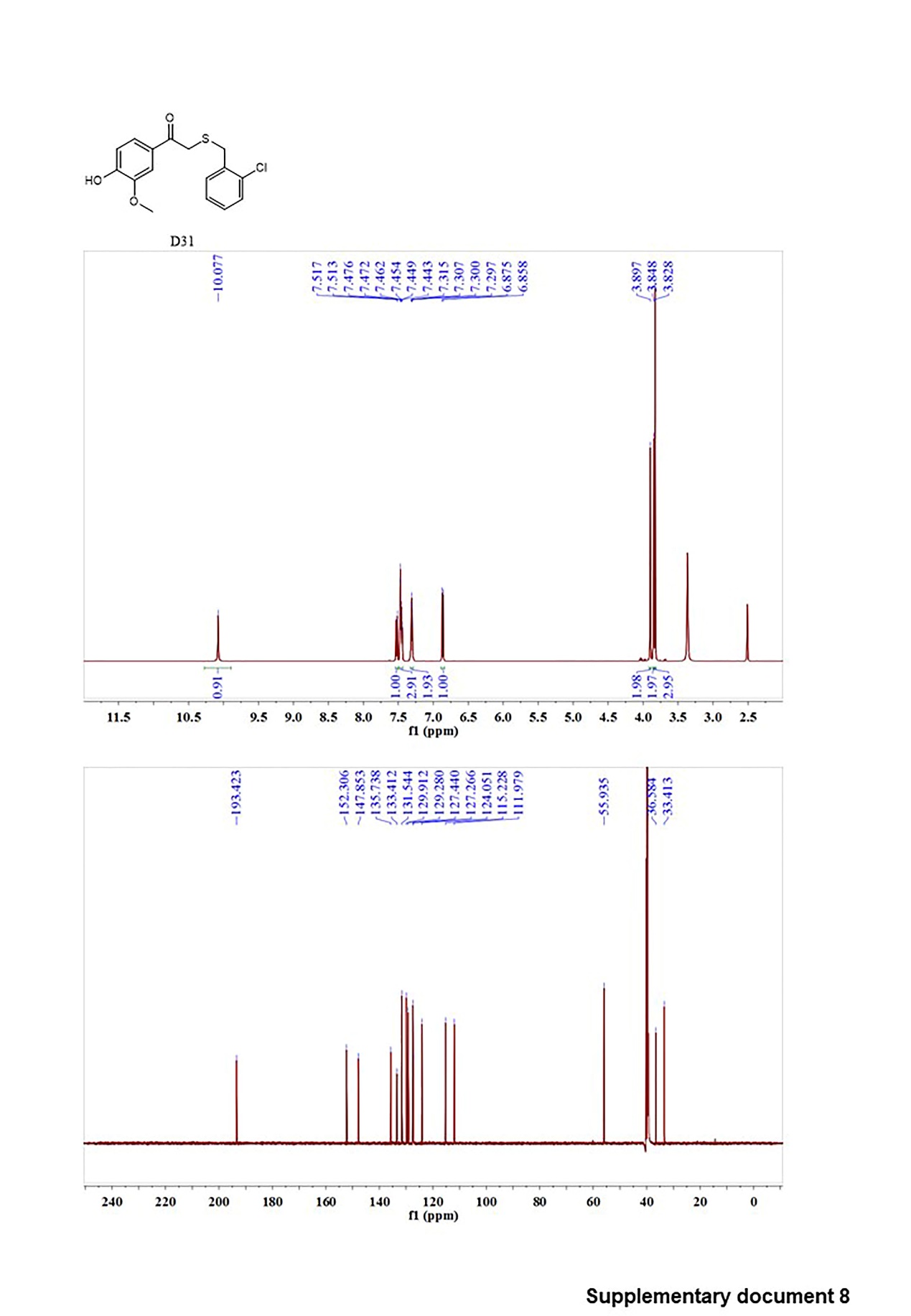


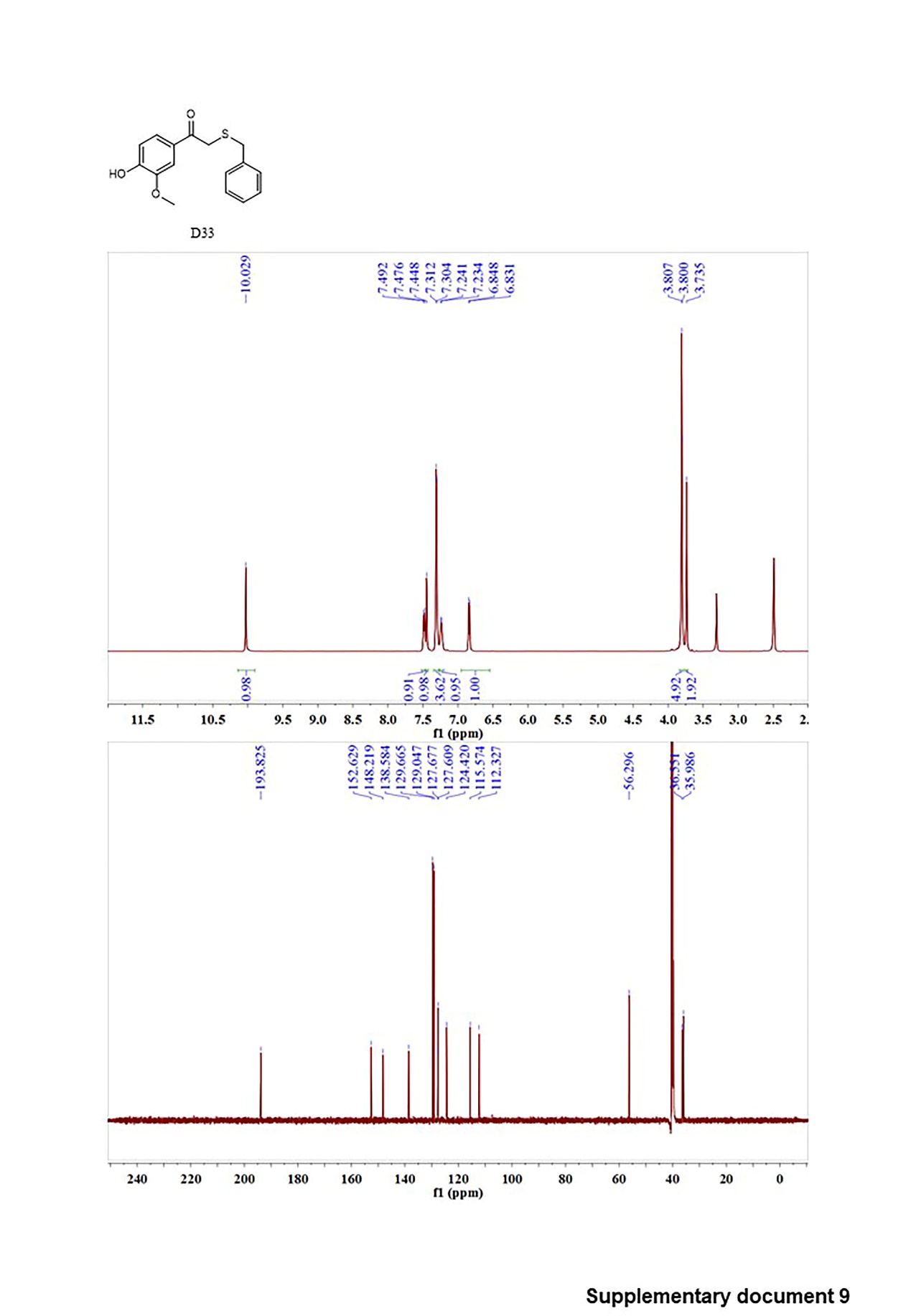


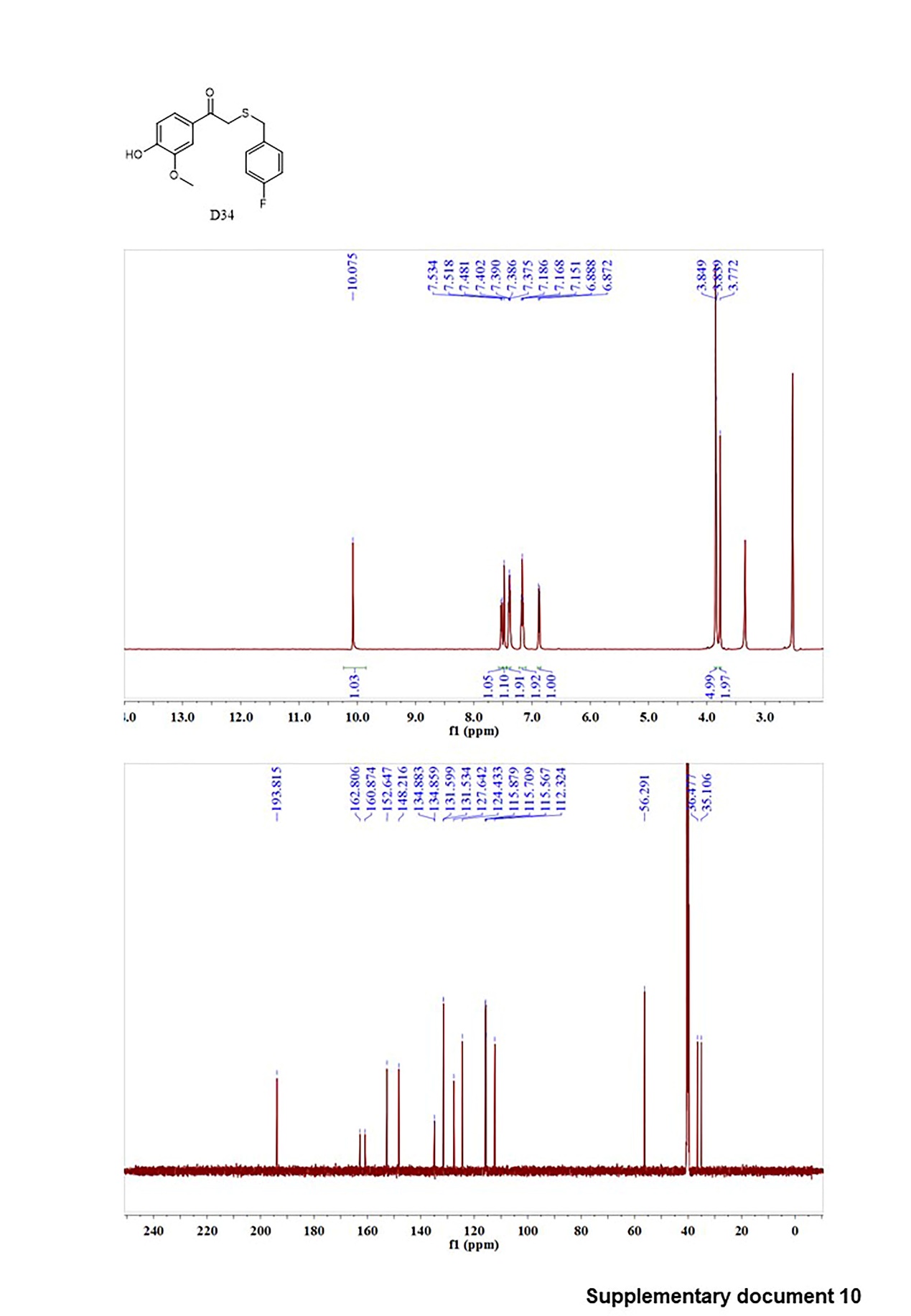

Supplement: Supplementary file 1 [file Table_1.docx]
